# Author Correction: Towards a data-integrated cell

**DOI:** 10.1038/s41467-019-10417-4

**Published:** 2019-05-21

**Authors:** Noël Malod-Dognin, Julia Petschnigg, Sam F. L. Windels, Janez Povh, Harry Hemingway, Robin Ketteler, Nataša Pržulj

**Affiliations:** 1 Department of Computer Science, University College London, London, WC1E 6BT UK; 2 Department of Life Science, Barcelona Supercomputing Center (BSC), Barcelona, 08034 Spain; 3 Faculty of Mechanical Engineering, University of Ljubljana, Ljubljana, 1000 Slovenia; 4 Health Data Research UK London, University College London, London, WC1E 6BT UK; 5 Institute of Health Informatics, University College London, London, WC1E 6BT UK; 6 The National Institute for Health Research University College London Hospitals Biomedical Research Centre, University College London, London, W1T 7DN UK; 7 MRC Laboratory for Molecular Cell Biology, University College London, London, WC1E 6BT UK; 8 ICREA, Pg. Lluís Companys 23, 08010 Barcelona, Spain

Correction to: *Nature Communications* 10.1038/s41467-019-08797-8, published online 18 February 2019.

The original version of this Article contained an error in the spelling of the author Harry Hemingway, which was incorrectly given as Harry Hemmingway. This has been corrected in both the PDF and HTML versions of the Article.

