# Supplementary material for: Towards a data-integrated cell
Source: Nat Commun. 2019 Feb 18;10:805. doi: 10.1038/s41467-019-08797-8 (PMC6379402; doi:10.1038/s41467-019-08797-8)
Supplement: Supplementary file 1 — Supplementary Information [file 41467_2019_8797_MOESM1_ESM.docx]

**Supplementary Information for: Towards a data-integrated cell**

Malod-Dognin et al.

This supplementary Information contains Supplementary Figures 1 to 11 and Supplementary Tables 1 to 6.

**Supplementary Figures**


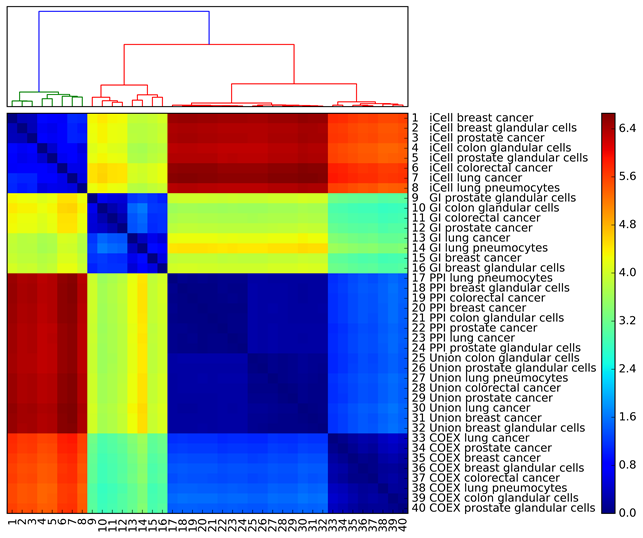


**Supplementary Figure 1**. **Structural similarities between networks.** The heat-map shows the GCD-11 distances between networks (listed in y-axis). “Union” denotes the simple union of interactions from PPI, COEX, and GI networks corresponding to a particular cancer or control tissue into one network. Distances close to 0 (dark blue) indicate structural similarity between networks.


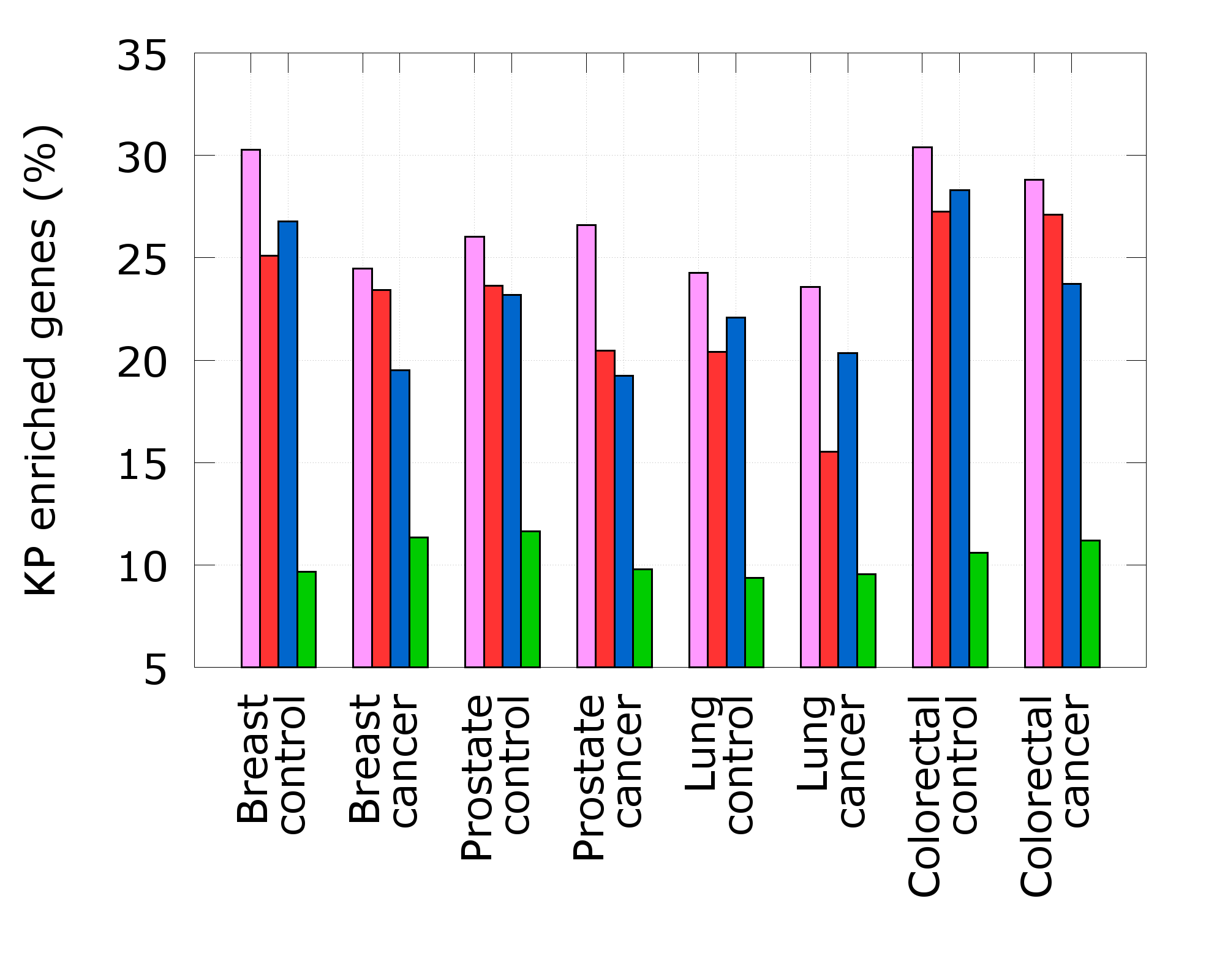


**Supplementary Figure 2. Kegg Pathway enrichments.** For the clusterings that are obtained for each tissue (x-axis) and for each of iCell (pink), PPI (red), COEX (blue) and GI (green) networks, the bars show the percentage of KEGG Pathway (KP) annotated genes having at least one annotation that is enriched in their clusters (y-axis).

**
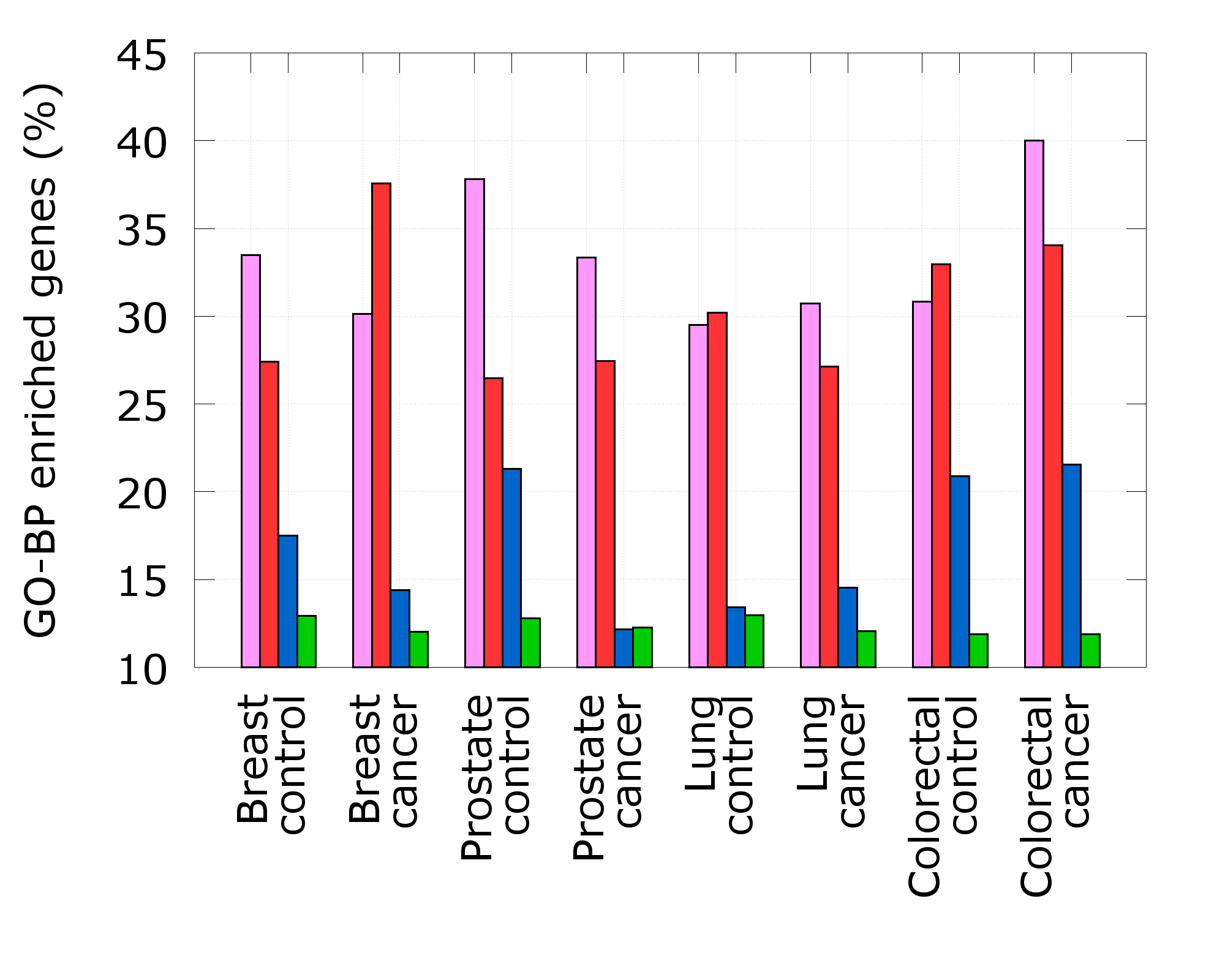
**

**Supplementary Figure 3. Gene Ontology biological process enrichments.** For the clusterings that are obtained for each tissue (x-axis) and for each of iCell (pink), PPI (red), COEX (blue) and GI (green) networks, the bars show the percentage of Gene Ontology Biological Process (GO-BP) annotated genes having at least one annotation that is enriched in their clusters (y-axis).


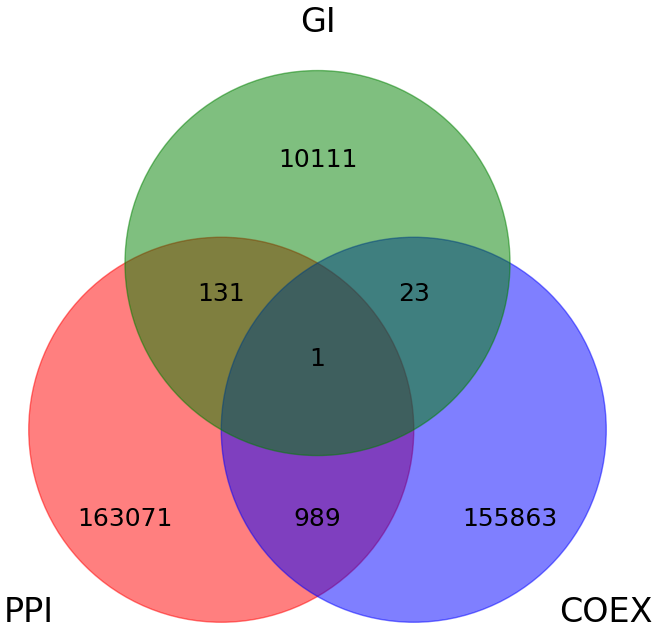

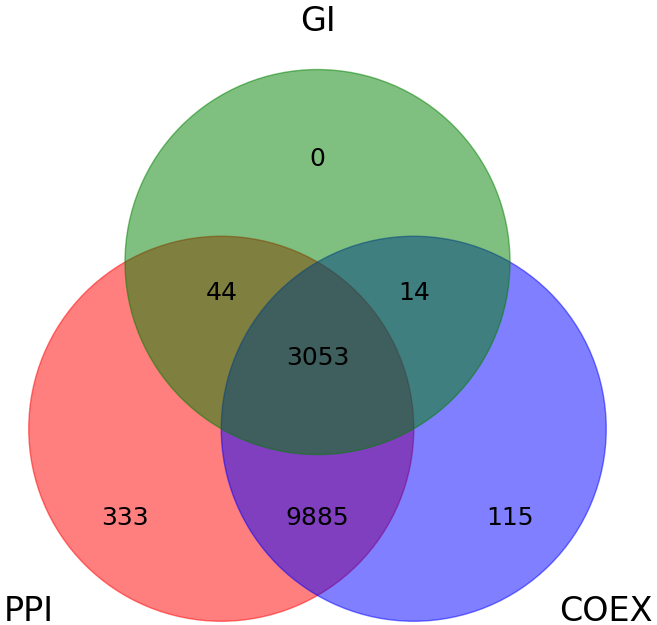


**Supplementary Figure 4.** **Overlap between the networks.** **Left**: The Venn diagram showing the overlap between the gene-sets of our human omics networks: protein-protein interaction (PPI), genetic interaction (GI), and co-expression (COEX) networks. **Right**: The Venn diagram showing the same, but in terms of the overlap between their interaction sets.


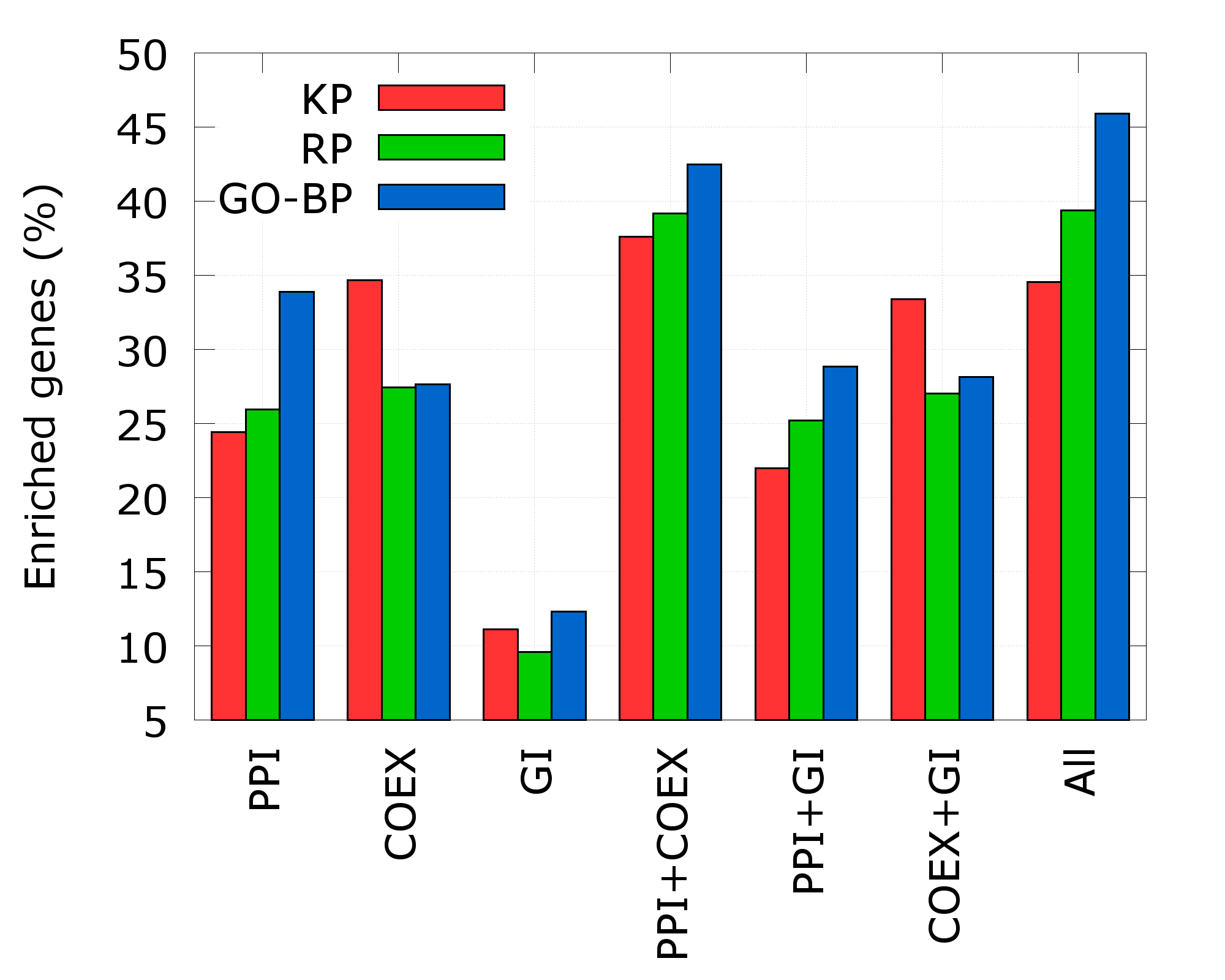


**Supplementary Figure 5. Benefit of integrating all datasets.** For each of the human PPI, COEX, and GI network, as well as for any of their combination, we created clusters of genes with our iCell methodology as detailed in the main document. For each clustering, the red bars show the percentage of the KEGG Pathway annotated genes having at least one annotation that is enriched in the clusters (KP). The green and blue bars show the same for Reactome pathway (RP) and Gene Ontology Biological Process (GO-BP) annotations, respectively.

**
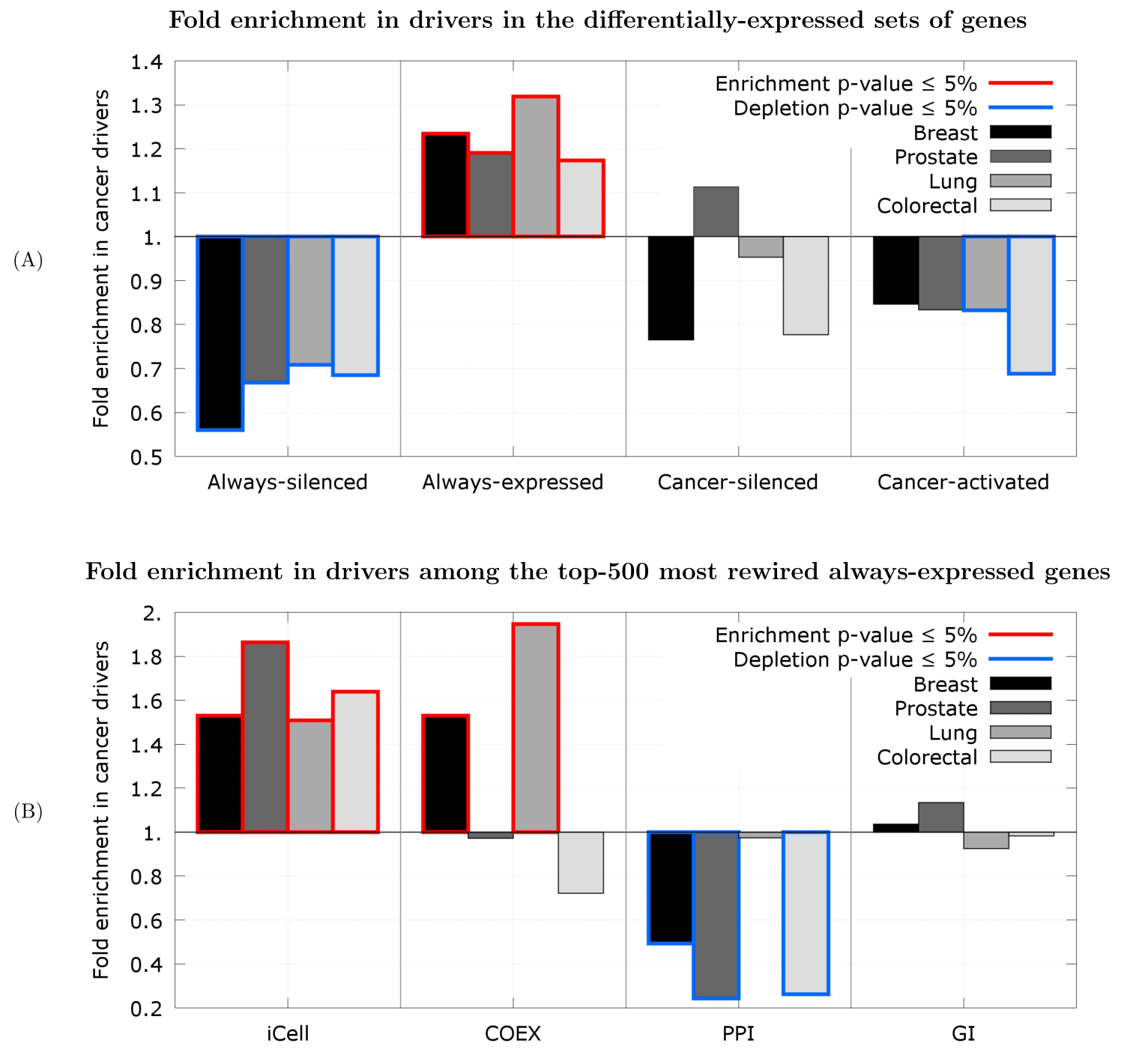
**

**Supplementary Figure 6. Cancer-specific study. A:** The fold enrichment in driver genes of the four sets of differentially-expressed genes, for each of the four cancers of interest. **B:** The fold enrichment in driver genes in the top 500 most rewired always-expressed genes in the iCell, COEX, PPI and GI networks for each of the four cancers of interest. In both panels, fold enrichment > 1 indicates enrichment in driver genes, fold enrichment < 1 indicates depletion in driver-genes, and statistically significant enrichments and depletions (with *p*-values ≤ 5%) are highlighted in red and blue, respectively.


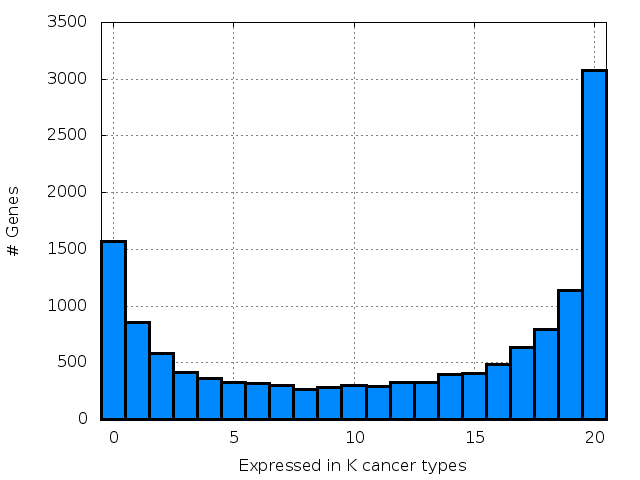


**Suplementary Figure 7.** **Pan-cancer gene expressions.** Number of genes (y-axis) that are expressed in exactly *K* different cancers (x-axis).


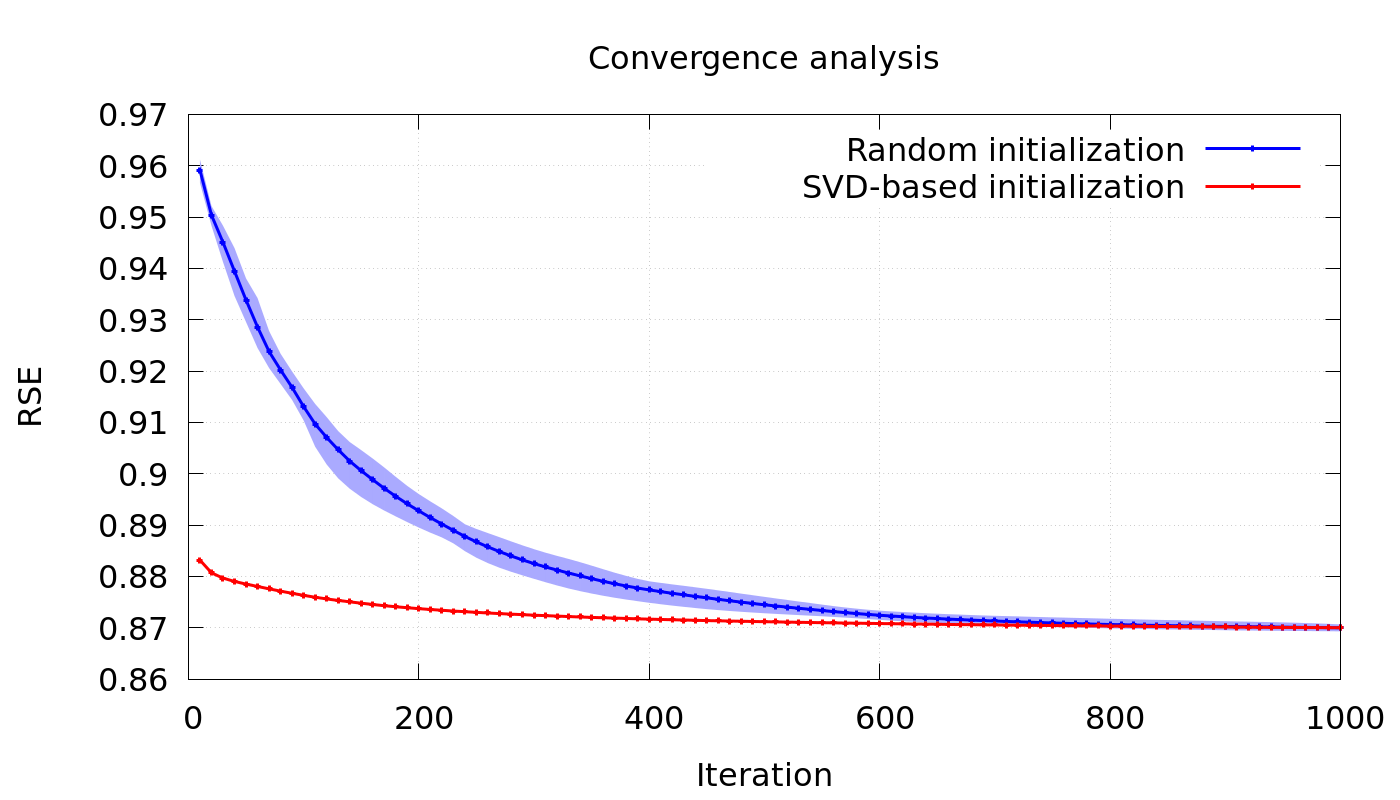


**Supplementary Figure 8**. **Convergence analysis of our integration framework.** Relative Square Errors (RSEs) that are obtained at different iterations of the fixed point solver when integrating the human PPI, COEX and GI networks. The red line shows the RSEs that are obtained when the solver is initialized with Singular Value Decomposition (SVD) based solution (for which the solver is deterministic). The blue line shows the average RSEs that are obtained over 10 runs in which the solver is initialized with different random solutions. The blue area around the line shows the minimum and maximum RSEs that are obtained in the 10 runs. For both SVD and random initializations, the number of clusters, *k*, is set to 50.


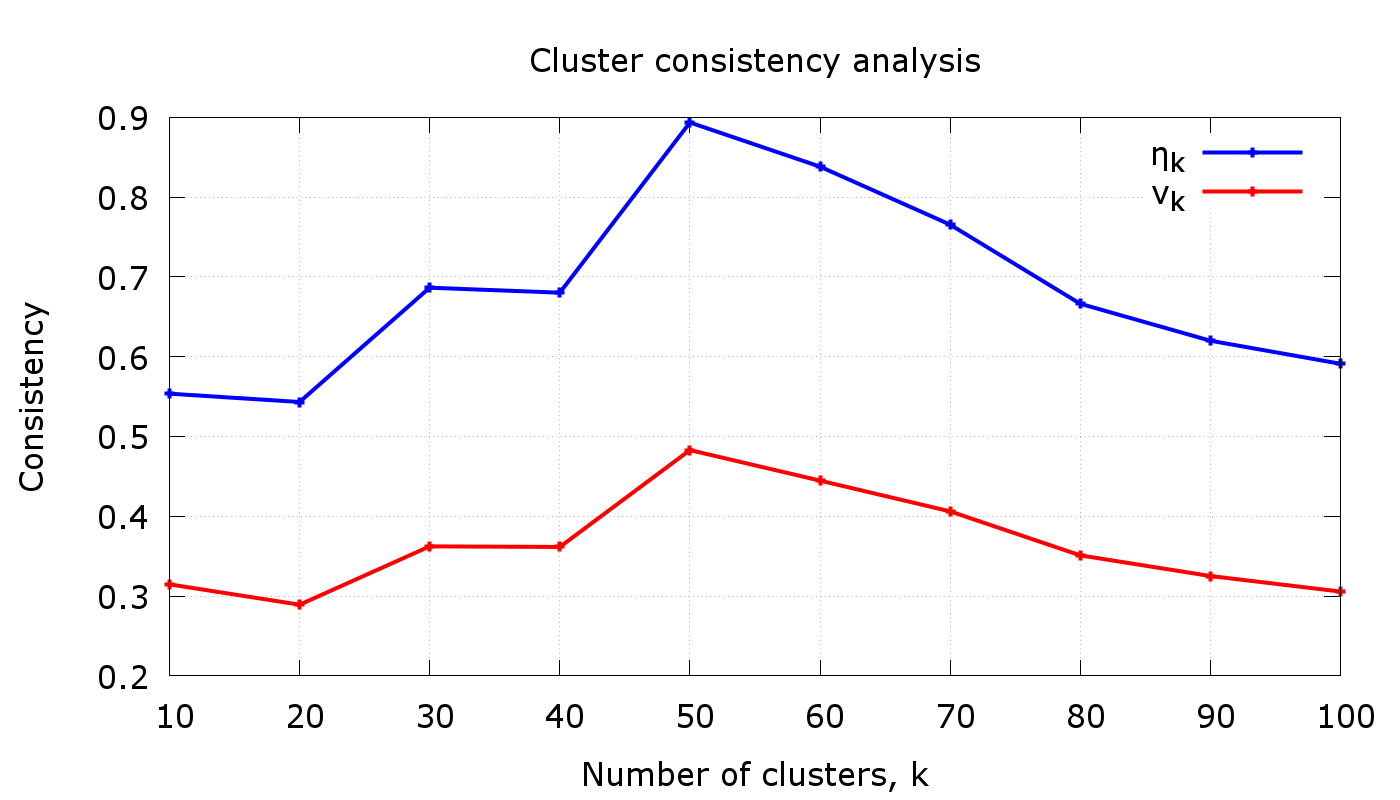


**Supplementary Figure 9. Cluster consistency analysis of our integration framework.** Consistency of 10 different clusterings that are obtained when integrating human PPI, COEX and GI networks, as a function of the number of clusters, *k*. For each *k* (varying from 10 to 100 in steps of 10), consistency is measured with dispersion coefficients $\eta_{k}$ and $\nu_{k}$ over 10 different runs in which the fixed point solver is initialized with different random solutions.


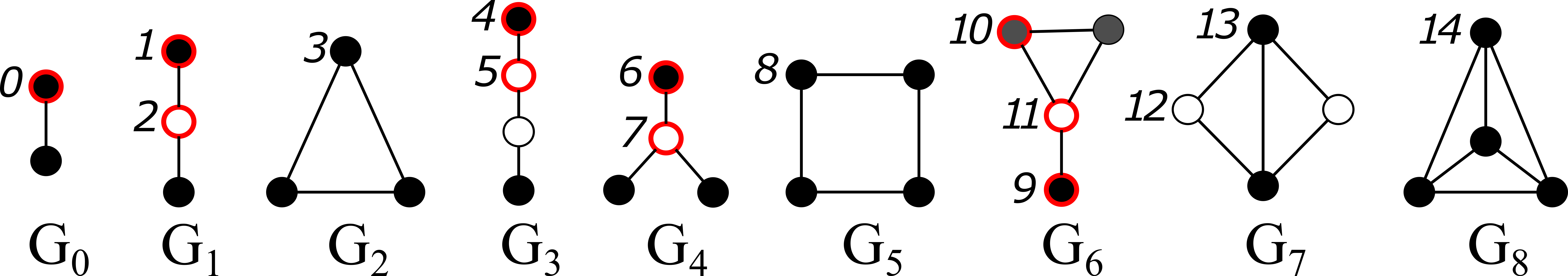


**Supplementary Figure 10.** **The nine 2- to 4-node graphlets and their 15 orbits.** Within each graphlet, nodes belonging to the same orbit have the same color (either white, black or grey). The ten non-redundant orbits, whose counts cannot be derived from the counts of the other orbits, are highlighted in red.


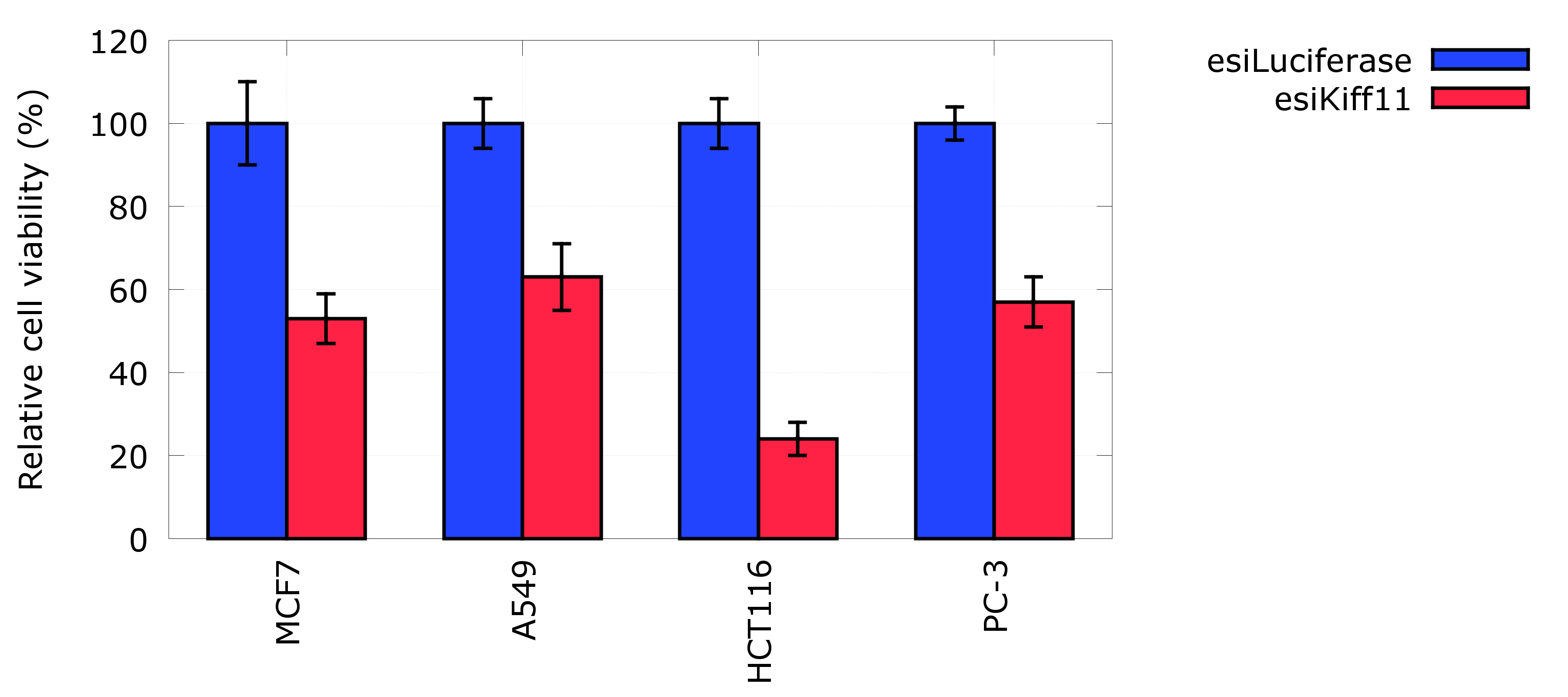


**Supplementary Figure 11.** **Control esiRNA transfections to assess knockdown efficiency**. Cancer cell lines (MCF7, A549, HCT116 and PC-3) were seeded in triplicates into 96-well plates and transfected with esiLuciferase or esiKif11 as controls. Three days after transfections, Presto blue cell viability assays were performed and cell viability changes upon Kif11-knockdown (in red) were normalized to esiLuciferase controls (in blue). The error-bars show the standard deviation over the triplicates.

**Supplementary Tables**

| **Breast cancer** | | **Prostate cancer** | |
| --- | --- | --- | --- |
| **Name** | **p-value** | **Name** | **p-value** |
| Vascular smooth muscle contraction | 3.71E-04 | Alcoholism | 5.80E-03 |
| Ovarian Steroidogenesis | 3.42E-03 | Long-term potentiation | 6.43E-03 |
| Estrogen signaling pathway | 5.08E-03 | Endocytosis | 6.70E-03 |
| Ribosome | 6.99E-03 | Insulin signaling pathway | 6.99E-03 |
| Inflammatory mediator regulation of TRP channels | 7.76E-03 | GnRH signaling pathway | 9.78E-03 |
| Epstein-Barr virus infection | 8.29E-03 | Bladder cancer | 1.29E-02 |
| GnRH signaling pathway | 9.78E-03 | VEGF signaling pathway | 1.41E-02 |
| Progesterone-mediated oocyte maturation | 1.05E-02 | Spliceosome | 1.58E-02 |
| Renin secretion | 1.68E-02 | Estrogen signaling pathway | 1.60E-02 |
| Ubiquitin mediated proteolysis | 1.96E-02 | Neurotrophin signaling pathway | 2.15E-02 |
| Colorectal cancer | 1.98E-02 | Longevity regulating pathway - multiple species | 2.15E-02 |
| Longevity regulating pathway - multiple species | 2.15E-02 | One carbon pool by folate | 2.18E-02 |
| Ras signaling pathway | 2.17E-02 | Viral carcinogenesis | 2.36E-02 |
| Legionellosis | 3.07E-02 | Non-alcoholic fatty liver disease (NAFLD) | 3.57E-02 |
| Ether lipid metabolism | 3.31E-02 | Tight junction | 3.57E-02 |
| Melanogenesis | 3.56E-02 | Antigen processing and presentation | 3.90E-02 |
| Endometrial cancer | 3.61E-02 | Chronic myeloid leukemia | 4.05E-02 |
| Chemokine signaling pathway | 3.77E-02 | Epstein-Barr virus infection | 4.10E-02 |
| Oocyte meiosis | 3.80E-02 | Choline metabolism in cancer | 4.94E-02 |
| RNA degradation | 4.32E-02 |  |  |
| Serotonergic synapse | 4.45E-02 |  |  |
| Parkinson's disease | 4.63E-02 |  |  |
| Olfactory transduction | 4.81E-02 |  |  |
| Purine metabolism | 4.82E-02 |  |  |
| VEGF signaling pathway | 4.85E-02 |  |  |
| **Lung cancer** | | **Colorectal cancer** | |
| **Name** | **p-value** | **Name** | **p-value** |
| mRNA surveillance pathway | 1.48E-02 | Non-small cell lung cancer | 8.92E-04 |
| Signaling pathways regulating pluripotency of stem cells | 1.89E-02 | Hepatitis B | 1.61E-03 |
| Ubiquitin mediated proteolysis | 2.29E-02 | Cell cycle | 2.04E-03 |
| Mucin type O-glycan biosynthesis | 2.85E-02 | Leishmaniasis | 2.11E-03 |
| Protein export | 2.85E-02 | Viral carcinogenesis | 6.24E-03 |
| Biosynthesis of vancomycin group | 3.39E-02 | Glioma | 8.37E-03 |
| Polyketide sugar unit | 3.39E-02 | MicroRNAs in cancer | 1.01E-02 |
| Sulfur relay system | 4.29E-02 | Neurotrophin signaling pathway | 1.08E-02 |
| Small cell lung cancer | 4.77E-02 | Amyotrophic lateral sclerosis (ALS) | 1.10E-02 |
|  |  | Endometrial cancer | 1.21E-02 |
|  |  | Bladder cancer | 1.57E-02 |
|  |  | Chronic myeloid leukemia | 1.65E-02 |
|  |  | Vitamin B6 metabolism | 2.20E-02 |
|  |  | Pantothenate and CoA biosynthesis | 2.48E-02 |
|  |  | Sphingolipid signaling pathway | 2.55E-02 |
|  |  | Renal cell carcinoma | 2.66E-02 |
|  |  | NF-kappa B signaling pathway | 3.11E-02 |
|  |  | RIG-I-like receptor signaling pathway | 3.33E-02 |
|  |  | Apoptosis | 3.37E-02 |
|  |  | Hepatitis C | 3.49E-02 |
|  |  | Legionellosis | 3.69E-02 |
|  |  | GnRH signaling pathway | 3.74E-02 |
|  |  | Prostate cancer | 4.21E-02 |
|  |  | Toll-like receptor signaling pathway | 4.21E-02 |
|  |  | FoxO signaling pathway | 4.24E-02 |
|  |  | Prolactin signaling pathway | 4.65E-02 |

**Supplementary Table 1.** The KEGG pathways that are enriched in the top 500 most rewired always-expressed genes in iCells.

| **Breast cancer** | | **Prostate cancer** | |
| --- | --- | --- | --- |
| **Name** | **p-value** | **Name** | **p-value** |
| Mitochondrial translation initiation | 9.01E-10 | mRNA Splicing - Major Pathway | 2.68E-06 |
| Mitochondrial translation termination | 9.01E-10 | HSF1 activation | 8.37E-04 |
| Mitochondrial translation elongation | 9.01E-10 | Lysosome Vesicle Biogenesis | 1.01E-03 |
| RNA Polymerase III Transcription Initiation From Type 3 Promoter | 1.07E-03 | Attenuation phase | 1.17E-03 |
| APC/C:Cdc20 mediated degradation of Cyclin B | 1.33E-03 | Negative regulation of MAPK pathway | 1.21E-03 |
| Rap1 signalling | 1.39E-03 | EGFR downregulation | 2.31E-03 |
| PKA-mediated phosphorylation of key metabolic factors | 4.03E-03 | Endosomal Sorting Complex Required For Transport (ESCRT) | 3.89E-03 |
| Gluconeogenesis | 4.55E-03 | HSF1-dependent transactivation | 4.31E-03 |
| Phosphorylation of the APC/C | 4.66E-03 | Vasopressin-like receptors | 4.33E-03 |
| RNA Polymerase III Abortive And Retractive Initiation | 5.27E-03 | VEGFR2 mediated cell proliferation | 5.30E-03 |
| Cytosolic sensors of pathogen-associated DNA | 5.66E-03 | Formation of TC-NER Pre-Incision Complex | 5.71E-03 |
| CD209 (DC-SIGN) signaling | 6.79E-03 | RAS signaling downstream of NF1 loss-of-function variants | 8.44E-03 |
| RNA Polymerase III Transcription Initiation From Type 2 Promoter | 6.79E-03 | Activation of RAS in B cells | 8.44E-03 |
| Lipid digestion, mobilization, and transport | 7.87E-03 | Transcription-Coupled Nucleotide Excision Repair (TC-NER) | 1.03E-02 |
| PKA-mediated phosphorylation of CREB | 7.87E-03 | Respiratory electron transport | 1.21E-02 |
| FGFR2 alternative splicing | 8.06E-03 | RAF activation | 1.25E-02 |
| RNA Polymerase III Transcription Initiation From Type 1 Promoter | 8.06E-03 | Eukaryotic Translation Elongation | 1.37E-02 |
| Ovarian tumor domain proteases | 8.92E-03 | Calmodulin induced events | 1.37E-02 |
| Regulation of APC/C activators between G1/S and early anaphase | 9.48E-03 | Budding and maturation of HIV virion | 1.44E-02 |
| APC-Cdc20 mediated degradation of Nek2A | 1.28E-02 | CDO in myogenesis | 1.65E-02 |
| Organic anion transporters | 1.28E-02 | SMAD2/SMAD3:SMAD4 heterotrimer regulates transcription | 1.88E-02 |
| Sulfide oxidation to sulfate | 1.28E-02 | Complex I biogenesis | 2.18E-02 |
| TFAP2A acts as a transcriptional repressor during retinoic acid induced cell differentiation | 1.28E-02 | N-glycan trimming in the ER and Calnexin/Calreticulin cycle | 2.19E-02 |
| Activation of Rac | 1.37E-02 | Ligand-independent caspase activation via DCC | 2.74E-02 |
| Synthesis of active ubiquitin: roles of E1 and E2 enzymes | 1.47E-02 | GRB2 events in EGFR signaling | 2.74E-02 |
| PKA activation in glucagon signalling | 1.66E-02 | SOS-mediated signalling | 2.74E-02 |
| PKA activation | 1.66E-02 | Constitutive Signaling by Ligand-Responsive EGFR Cancer Variants | 3.01E-02 |
| MAP3K8 (TPL2)-dependent MAPK1/3 activation | 1.66E-02 | activated TAK1 mediates p38 MAPK activation | 3.01E-02 |
| VEGFA-VEGFR2 Pathway | 1.86E-02 | JNK (c-Jun kinases) phosphorylation and activation mediated by activated human TAK1 | 3.01E-02 |
| CREB phosphorylation through the activation of Adenylate Cyclase | 1.87E-02 | mRNA Splicing - Minor Pathway | 3.10E-02 |
| TNFR1-induced NFkappaB signaling pathway | 1.90E-02 | Translesion Synthesis by POLH | 3.48E-02 |
| RNA Polymerase III Chain Elongation | 1.99E-02 | SHC1 events in ERBB2 signaling | 3.48E-02 |
| Regulation of PLK1 Activity at G2/M Transition | 2.24E-02 | HuR (ELAVL1) binds and stabilizes mRNA | 3.56E-02 |
| TP53 Regulates Transcription of Genes Involved in G2 Cell Cycle Arrest | 2.35E-02 | Role of Abl in Robo-Slit signaling | 3.56E-02 |
| PI3K/AKT activation | 2.56E-02 | SHC1 events in EGFR signaling | 3.56E-02 |
| Nef mediated downregulation of MHC class I complex cell surface expression | 2.56E-02 | Pyrimidine salvage reactions | 3.56E-02 |
| Regulation of TNFR1 signaling | 2.67E-02 | Endosomal/Vacuolar pathway | 3.56E-02 |
| Interleukin-1 signaling | 2.67E-02 | Amine ligand-binding receptors | 3.86E-02 |
| Interleukin-3, 5 and GM-CSF signaling | 2.74E-02 | TWIK related potassium channel (TREK) | 3.86E-02 |
| Conversion from APC/C:Cdc20 to APC/C:Cdh1 in late anaphase | 3.17E-02 | Cytochrome P450 - arranged by substrate type | 3.86E-02 |
| Fatty Acyl-CoA Biosynthesis | 3.17E-02 | Regulation of FZD by ubiquitination | 3.98E-02 |
| Branched-chain amino acid catabolism | 3.17E-02 | Tight junction interactions | 3.98E-02 |
| DNA Damage Recognition in GG-NER | 3.28E-02 | Mitochondrial translation initiation | 4.03E-02 |
| Recycling of eIF2:GDP | 3.33E-02 | Processing of Capped Intron-Containing Pre-mRNA | 4.43E-02 |
| Inactivation of Cdc42 and Rac | 3.33E-02 | SHC-related events triggered by IGF1R | 4.46E-02 |
| APC/C:Cdc20 mediated degradation of mitotic proteins | 3.63E-02 | Signaling by FGFR3 fusions in cancer | 4.46E-02 |
| RNA Polymerase III Transcription Termination | 3.63E-02 | Insulin receptor signalling cascade | 4.46E-02 |
| Tight junction interactions | 3.63E-02 | DAP12 interactions | 4.46E-02 |
| Inactivation of APC/C via direct inhibition of the APC/C complex | 3.63E-02 | IRAK2 mediated activation of TAK1 complex | 4.46E-02 |
| TRKA activation by NGF | 3.72E-02 | IRAK2 mediated activation of TAK1 complex upon TLR7/8 or 9 stimulation | 4.46E-02 |
| APC truncation mutants are not K63 polyubiquitinated | 3.72E-02 | Signalling to RAS | 4.46E-02 |
| Processing of Capped Intron-Containing Pre-mRNA | 3.97E-02 | EGFR Transactivation by Gastrin | 4.46E-02 |
| Hedgehog 'off' state | 4.00E-02 | PTK6 Regulates RTKs and Their Effectors AKT1 and DOK1 | 4.46E-02 |
| Anchoring of the basal body to the plasma membrane | 4.04E-02 | Gap-filling DNA repair synthesis and ligation in TC-NER | 4.89E-02 |
| Regulation of signaling by CBL | 4.12E-02 |  |  |
| Association of TriC/CCT with target proteins during biosynthesis | 4.34E-02 |  |  |
| Synthesis of PC | 4.65E-02 |  |  |
| **Lung cancer** | | **Colorectal cancer** | |
| **Name** | **p-value** | **Name** | **p-value** |
| Reversal of alkylation damage by DNA dioxygenases | 1.11E-03 | RAS signaling downstream of NF1 loss-of-function variants | 2.81E-04 |
| MAPK1 (ERK2) activation | 1.82E-03 | Mitochondrial translation elongation | 6.24E-04 |
| Establishment of Sister Chromatid Cohesion | 3.71E-03 | p38MAPK events | 1.14E-03 |
| Cohesin Loading onto Chromatin | 3.71E-03 | Electric Transmission Across Gap Junctions | 1.74E-03 |
| POU5F1 (OCT4), SOX2, NANOG activate genes related to proliferation | 4.97E-03 | Regulation of TP53 Activity through Phosphorylation | 1.90E-03 |
| Josephin domain DUBs | 4.97E-03 | GRB2 events in EGFR signaling | 2.23E-03 |
| Antigen processing: Ubiquitination & Proteasome degradation | 5.84E-03 | SOS-mediated signalling | 2.23E-03 |
| Signaling by MST1 | 6.37E-03 | Mitochondrial translation initiation | 2.28E-03 |
| Gap junction assembly | 7.61E-03 | Mitochondrial translation termination | 2.28E-03 |
| Downstream TCR signaling | 8.29E-03 | mRNA Splicing - Minor Pathway | 2.74E-03 |
| RNA Polymerase II Pre-transcription Events | 8.50E-03 | SHC1 events in EGFR signaling | 3.46E-03 |
| RNA polymerase II transcribes snRNA genes | 1.24E-02 | Coenzyme A biosynthesis | 3.46E-03 |
| Elongation arrest and recovery | 1.24E-02 | N-glycan trimming in the ER and Calnexin/Calreticulin cycle | 3.66E-03 |
| Negative feedback regulation of MAPK pathway | 1.52E-02 | SHC-related events triggered by IGF1R | 5.04E-03 |
| Molybdenum cofactor biosynthesis | 1.52E-02 | Signaling by FGFR3 fusions in cancer | 5.04E-03 |
| Constitutive Signaling by NOTCH1 PEST Domain Mutants | 1.79E-02 | Stabilization of p53 | 5.04E-03 |
| Constitutive Signaling by NOTCH1 HD+PEST Domain Mutants | 1.79E-02 | Insulin receptor signalling cascade | 5.04E-03 |
| Interleukin-1 signaling | 1.88E-02 | Signalling to RAS | 5.04E-03 |
| Chondroitin sulfate biosynthesis | 2.07E-02 | EGFR Transactivation by Gastrin | 5.04E-03 |
| Signaling by NOTCH3 | 2.09E-02 | Constitutive Signaling by Ligand-Responsive EGFR Cancer Variants | 5.76E-03 |
| UCH proteinases | 2.44E-02 | Signaling by FGFR4 in disease | 6.98E-03 |
| RNA Polymerase II Transcription Elongation | 2.49E-02 | Establishment of Sister Chromatid Cohesion | 6.98E-03 |
| Hedgehog 'on' state | 2.54E-02 | MET activates RAS signaling | 6.98E-03 |
| Downregulation of ERBB4 signaling | 2.72E-02 | Cohesin Loading onto Chromatin | 6.98E-03 |
| tRNA modification in the mitochondrion | 2.72E-02 | Activation of the AP-1 family of transcription factors | 6.98E-03 |
| Nephrin interactions | 2.76E-02 | Josephin domain DUBs | 9.30E-03 |
| Formation of RNA Pol II elongation complex | 2.89E-02 | SHC-mediated cascade:FGFR3 | 9.30E-03 |
| Hh mutants that don't undergo autocatalytic processing are degraded by ERAD | 3.34E-02 | Lysine catabolism | 9.30E-03 |
| Defective SLCO1B1 causes hyperbilirubinemia, Rotor type (HBLRR) | 3.34E-02 | SHC1 events in ERBB4 signaling | 9.30E-03 |
| Defective SLCO1B3 causes hyperbilirubinemia, Rotor type (HBLRR) | 3.34E-02 | Transport and synthesis of PAPS | 9.88E-03 |
| Activation of PUMA and translocation to mitochondria | 3.42E-02 | Activation of RAS in B cells | 9.88E-03 |
| MAPK3 (ERK1) activation | 3.42E-02 | Regulation of TP53 Degradation | 1.12E-02 |
| IRAK2 mediated activation of TAK1 complex | 3.42E-02 | DNA Damage Recognition in GG-NER | 1.12E-02 |
| IRAK2 mediated activation of TAK1 complex upon TLR7/8 or 9 stimulation | 3.42E-02 | SHC-mediated cascade:FGFR1 | 1.20E-02 |
| ATF6 (ATF6-alpha) activates chaperone genes | 3.42E-02 | GRB2 events in ERBB2 signaling | 1.51E-02 |
| FCERI mediated NF-kB activation | 3.45E-02 | FRS-mediated FGFR3 signaling | 1.51E-02 |
| CLEC7A (Dectin-1) signaling | 3.65E-02 | Transport of nucleotide sugars | 1.60E-02 |
| Hedgehog ligand biogenesis | 3.82E-02 | Signaling by FGFR1 in disease | 1.64E-02 |
| Interleukin-6 signaling | 4.19E-02 | FRS-mediated FGFR1 signaling | 1.87E-02 |
| NOTCH1 Intracellular Domain Regulates Transcription | 4.33E-02 | Signaling by FGFR3 point mutants in cancer | 1.87E-02 |
| RAF activation | 4.44E-02 | FRS-mediated FGFR4 signaling | 1.87E-02 |
| Insulin processing | 4.44E-02 | Constitutive Signaling by EGFRvIII | 1.87E-02 |
| Defective CFTR causes cystic fibrosis | 4.63E-02 | DAP12 signaling | 2.16E-02 |
| EGFR downregulation | 4.92E-02 | Formation of Incision Complex in GG-NER | 2.22E-02 |
|  |  | SHC-mediated cascade:FGFR4 | 2.27E-02 |
|  |  | Oxidative Stress Induced Senescence | 2.36E-02 |
|  |  | Oncogene Induced Senescence | 2.46E-02 |
|  |  | Regulation of TP53 Activity through Methylation | 2.70E-02 |
|  |  | Tie2 Signaling | 2.70E-02 |
|  |  | FRS-mediated FGFR2 signaling | 2.70E-02 |
|  |  | Activation of PPARGC1A (PGC-1alpha) by phosphorylation | 3.18E-02 |
|  |  | Synthesis of 5-eicosatetraenoic acids | 3.18E-02 |
|  |  | Nicotinamide salvaging | 3.18E-02 |
|  |  | Signaling by FGFR2 in disease | 3.18E-02 |
|  |  | SHC-mediated cascade:FGFR2 | 3.18E-02 |
|  |  | ERK/MAPK targets | 3.18E-02 |
|  |  | activated TAK1 mediates p38 MAPK activation | 3.71E-02 |
|  |  | Chondroitin sulfate biosynthesis | 3.71E-02 |
|  |  | Sterols are 12-hydroxylated by CYP8B1 | 4.18E-02 |
|  |  | Defective MTRR causes methylmalonic aciduria and homocystinuria type cblE | 4.18E-02 |
|  |  | Defective MTR causes methylmalonic aciduria and homocystinuria type cblG | 4.18E-02 |
|  |  | Translesion Synthesis by POLH | 4.27E-02 |
|  |  | SHC1 events in ERBB2 signaling | 4.27E-02 |
|  |  | FCERI mediated MAPK activation | 4.29E-02 |

**Supplementary Table 2.** The Reactome pathways that are enriched in the top-500 most rewired always-expressed genes in iCells

| **Breast cancer (BRCA, T=1085, PC=112)** | | |
| --- | --- | --- |
| **Gene** | **Differential expression** | **Adjusted p-value** |
| CCNB1 | 2.982 | 9.81E-209 |
| HLA-DQA2 | 1.283 | 3.36E-17 |
| MRPL42 | 1.054 | 2.76E-78 |
| MRPL3 | 1.008 | 2.66E-100 |
| CD300LG | -5.376 | 0.00E+00 |
|  |  |  |
| **Prostate cancer (PRAD, T=492, PC=52)** | | |
| **Gene** | **Differential expression** | **Adjusted p-value** |
| MAZ | 1.354 | 2.69E-72 |
| TNXB | -1.345 | 6.56E-26 |
| KANK2 | -1.435 | 6.79E-28 |
| ARHGAP23 | -1.7 | 4.12E-41 |
|  |  |  |
| **Lung cancer (LUAD, T=483, PC=59)** | | |
| **Gene** | **Differential expression** | **Adjusted p-value** |
| RBM25 | -1.015 | 1.04E-37 |
| ANKZF1 | -1.134 | 6.68E-25 |
|  |  |  |
| **Colorectal cancer (COAD, T=275, PC=41)** | | |
| **Gene** | **Differential expression** | **Adjusted p-value** |
| CLDN4 | 4.669 | 2.98E-42 |
| TMPRSS4 | 3.718 | 5.93E-40 |
| PLEKHN1 | 1.334 | 2.17E-95 |
| HTR4 | -1.217 | 2.64E-58 |
| ADARB1 | -2.237 | 5.17E-47 |
| CNR1 | -2.654 | 5.98E-99 |

**Supplementary Table 3.** For each of the breast, prostate, lung, and colorectal cancers, we report the genes that our new methodology prioritized that are significantly differentially expressed in cancer tissues (T) with respect to the paired control tissues (PC), with ANOVA adjusted p-values ≤ 5%.

|  | **Network statistics** | | | | | |
| --- | --- | --- | --- | --- | --- | --- |
|  | **PPI** | | **COEX** | | **GI** | |
| **Tissue** | **#Node** | **#Edge** | **#Node** | **#Edge** | **#Node** | **#Edge** |
| Breast control | 9,188 | 106,198 | 9,233 | 84,930 | 2,269 | 4,998 |
| Prostate control | 8,963 | 97,699 | 9,051 | 81,649 | 2,189 | 5,543 |
| Lung control | 6,753 | 63,087 | 7,022 | 50,184 | 1,658 | 3,204 |
| Colon control | 10,257 | 120,851 | 10,263 | 103,106 | 2,487 | 6,766 |
| Breast cancer * | 8,260 | 93,416 | 8,378 | 74,147 | 2,027 | 4,679 |
| Carcinoid | 8,064 | 85,693 | 8,242 | 69,852 | 1,981 | 4,603 |
| Cervical cancer | 7,137 | 77,122 | 7,303 | 58,874 | 1,790 | 3,984 |
| Colorectal cancer * | 8,760 | 100,196 | 8,844 | 80,902 | 2,206 | 5,981 |
| Endometrial cancer | 7,632 | 82,061 | 7,788 | 64,467 | 1,825 | 4,210 |
| Glioma | 6,467 | 68,374 | 6,672 | 48,599 | 1,464 | 2,826 |
| Head and neck cancer | 8,446 | 97,078 | 8,554 | 75,823 | 2,154 | 5,440 |
| Liver cancer | 7,632 | 75,625 | 7,833 | 63,646 | 1,843 | 4,253 |
| Lung cancer * | 6,839 | 70,437 | 6,980 | 53,857 | 1,738 | 3,724 |
| Lymphoma | 5,373 | 53,498 | 5,599 | 38,831 | 1,363 | 2,693 |
| Melanoma | 7,672 | 83,714 | 7,818 | 65,731 | 1,884 | 3,856 |
| Ovarian cancer | 7,915 | 86,299 | 8,065 | 69,074 | 1,937 | 4,326 |
| Pancreatic cancer | 8,187 | 89,535 | 8,300 | 71,938 | 1,976 | 4,947 |
| Prostate cancer * | 7,675 | 79,969 | 7,851 | 64,625 | 1,890 | 5,122 |
| Renal cancer | 5,983 | 52,481 | 6,237 | 41,114 | 1,459 | 2,982 |
| Skin cancer | 6,549 | 70,117 | 6,719 | 51,275 | 1,683 | 3,586 |
| Stomach cancer | 7,409 | 79,488 | 7,575 | 62,078 | 1,866 | 4,585 |
| Testis cancer | 7,127 | 78,498 | 7,269 | 58,912 | 1,760 | 3,793 |
| Thyroid cancer | 9,213 | 104,463 | 9,301 | 86,323 | 2,256 | 5,951 |
| Urothelial cancer | 7,733 | 86,519 | 7,852 | 66,547 | 1,952 | 4,080 |

**Supplementary Table 4. Sizes of the tissue-specific networks used in the study.** For each tissue, columns report the numbers of nodes and edges of the corresponding tissue-specific protein-protein interaction (PPI), co-expression (COEX), and genetic interaction (GI) networks. Stars indicate the cancer tissues that we used in the cancer-specific studies.

|  |  | **Functional domain enrichments** | |  |  |
| --- | --- | --- | --- | --- | --- |
|  |  | **Avg. domain counts** | | **Empirical *p*-values** | |
| **Annotation** | **Tissue** | **iCell (10 runs)** | **randomized iCells**  **(1,000 runs)** | **Lowerbound** | **Upperbound** |
| BP | breast control | 9.800 | 0.291 | 0.001 | 0.001 |
| BP | breast cancer | 8.200 | 0.202 | 0.001 | 0.001 |
| BP | colorectal control | 10.000 | 0.340 | 0.001 | 0.001 |
| BP | colorectal cancer | 8.300 | 0.266 | 0.001 | 0.001 |
| BP | lung control | 6.100 | 0.156 | 0.001 | 0.001 |
| BP | lung cancer | 5.800 | 0.164 | 0.001 | 0.001 |
| BP | prostate control | 11.600 | 0.297 | 0.001 | 0.001 |
| BP | prostate cancer | 7.500 | 0.219 | 0.001 | 0.001 |
| CC | breast control | 12.400 | 0.484 | 0.001 | 0.001 |
| CC | breast cancer | 12.900 | 0.397 | 0.001 | 0.001 |
| CC | colorectal control | 13.400 | 0.558 | 0.001 | 0.001 |
| CC | colorectal cancer | 11.200 | 0.484 | 0.001 | 0.001 |
| CC | lung control | 8.800 | 0.282 | 0.001 | 0.002 |
| CC | lung cancer | 8.500 | 0.338 | 0.001 | 0.001 |
| CC | prostate control | 13.600 | 0.506 | 0.001 | 0.001 |
| CC | prostate cancer | 12.200 | 0.407 | 0.001 | 0.001 |
| MF | breast control | 5.800 | 0.350 | 0.001 | 0.002 |
| MF | breast cancer | 5.400 | 0.250 | 0.001 | 0.001 |
| MF | colorectal control | 7.400 | 0.317 | 0.001 | 0.001 |
| MF | colorectal cancer | 6.700 | 0.270 | 0.001 | 0.001 |
| MF | lung control | 3.400 | 0.184 | 0.001 | 0.001 |
| MF | lung cancer | 3.800 | 0.207 | 0.001 | 0.002 |
| MF | prostate control | 5.400 | 0.314 | 0.001 | 0.001 |
| MF | prostate cancer | 4.000 | 0.242 | 0.001 | 0.005 |
| KP | breast control | 14.100 | 1.155 | 0.001 | 0.001 |
| KP | breast cancer | 14.700 | 0.993 | 0.001 | 0.001 |
| KP | colorectal control | 16.400 | 1.417 | 0.001 | 0.001 |
| KP | colorectal cancer | 13.800 | 1.009 | 0.001 | 0.001 |
| KP | lung control | 11.700 | 0.737 | 0.001 | 0.001 |
| KP | lung cancer | 7.200 | 0.728 | 0.001 | 0.003 |
| KP | prostate control | 12.000 | 1.175 | 0.001 | 0.001 |
| KP | prostate cancer | 13.700 | 0.883 | 0.001 | 0.001 |
| RP | breast control | 13.400 | 0.617 | 0.001 | 0.001 |
| RP | breast cancer | 14.700 | 0.616 | 0.001 | 0.001 |
| RP | colorectal control | 15.700 | 0.698 | 0.001 | 0.001 |
| RP | colorectal cancer | 13.100 | 0.605 | 0.001 | 0.001 |
| RP | lung control | 10.000 | 0.402 | 0.001 | 0.001 |
| RP | lung cancer | 7.800 | 0.437 | 0.001 | 0.001 |
| RP | prostate control | 14.900 | 0.643 | 0.001 | 0.001 |
| RP | prostate cancer | 13.900 | 0.477 | 0.001 | 0.001 |

**Supplementary Table 5.** **Functional domain enrichments for the cancer and control iCells.** BP, CC, and MF stand for Biological Process, Cellular Component, and Molecular Function annotations from Gene Ontology, while KP stands for KEGG Pathways and RP for REACTOME Pathways annotations.

| **Gene or transcript IDs** | **Gene name** | **Sequence** |
| --- | --- | --- |
| ENSG00000107331, NM_212533, NM_001606 | ABCA2 | ATCTCTTCATCGGCATCACCGCCACCGTGGCCACCTTCCTGCTACAGCTCTTCGAGCACGACAAGGACCTGAAGGTTGTCAACAGTTACCTGAAAAGCTGCTTCCTCATTTTCCCCAACTACAACCTGGGCCACGGGCTCATGGAGATGGCCTACAACGAGTACATCAACGAGTACTACGCCAAGATTGGCCAGTTTGACAAGATGAAGTCCCCGTTCGAGTGGGACATTGTCACCCGCGGACTGGTGGCCATGGCGGTTGAGGGCGTCGTGGGCTTCCTCCTGACCATCATGTGCCAGTACAACTTCCTGCGGCGGCCACAGCGCATGCCTGTGTCTACCAAGCCTGTGGAGGATGATGTGGACGTGGCCAGTGAGCGGCAGCGAGTGCTCCGGGGAGACGCCGACAATGACAT |
| ENSG00000182827, NM_022735 | ACBD3 | AACGTCTGCAAAAGGAGGAAGAGAAACGTAGGAGAGAAGAAGAGGAAAGGCTTCGACGGGAGGAAGAGGAAAGGAGACGGATAGAAGAAGAAAGGCTTCGGTTGGAGCAGCAAAAGCAGCAGATAATGGCAGCTTTAAACTCCCAGACTGCCGTGCAGTTCCAGCAGTATGCAGCCCAACAGTATCCAGGGAACTACGAACAGCAGCAAATTCTCATCCGCCAGTTGCAGGAGCAACACTATCAGCAGTACATGCAGCAGTTGTATCAAGTCCAGCTTGCACAGCAACAGGCAGCATTACAGAAACAACAGGAAGTAGTAGTGGCTGGGTCTTCCTTGCCTACATCATCAAAAGTGAATGCAACTGTACCAAGTAATATGATGTCAGTTAATGGACAGGCCAAAACACACACTGACAGCTCCGAAAAAGAACTGGAACCAGAAGCTGC |
| ENSG00000197381, NM_001112, NM_015833, NM_001033049, NM_015834 | ADARB1 | AATCACGAGGGCTACTGCACAATACATGGCCTAAGTTCCCTCTGTTCCTTCCTCTGAATCGAATGGATGTGGGTGACCGCCCGAAGGCCTTCACAGGATGGAAGTAGAATGATTTCAGTAGATACTCATTCTTGGAAAATGCCATAGTTTTAAATTATTGTTTCCAGCTTTATCAAAGACATGTTTGAAAAATAAAAAGCATCCAAGTGAGAGCTGGTGAGACCACGTGCTGCTGGCGTAGTGTAGGCCAGACATTGACAGTCCTGACGGGAGCTCAGGGCTGCCCAGCGCCCAGCGTGCACGGGACGGCCCCACGACAGAGGGAGTCAGCC |
| ENSG00000101901, NM_018466 | ALG13 | CGTGCAGAGCCAGACTATGAAACTTCAGGTGTTTATAGCACAACTGCATCTACAGCAAACTTGTCTCTTCAGGACAGAAAGTCATGTTCTATGTCTCCTCAGGACACAGTTACCTCATACAACTACCCCCAGAAGATGATGGGAAATATTGCAGCAGTTGCAGCTTCCTGTGCCAATAATGTTCCAGCTCCAGTCTTATCTAACGGTGCAGCGGCTAATCAAGCTATTAGTACCACTTCAGTTTCCTCACAGAATGCTATACAGCCTCTCTTTGTATCTCCACCTACACACGGCAGGCCAGATACAAAAGTTTTGCAGTACTATTTCAATCTAGGATTGCAGTGCTATTACCACAGCTACTGGCACTCCATGGTCTATGTGCCACAGATGCAGCAGCAGCTTCATGTAGAGAATTATCCAGTCTATACTGAGCCACCTCTGGTAGATCAAACCGTTCCTCAATGC |
| ENSG00000166295, NM_173473 | ANAPC16 | CCAAAGGAGCTGGAGAGATGTTAGAAGATGGCTCTGAGAGATTCCTCTGCGAATCTGTTTTTAGCTATCAAGTGGCATCCACGCTTAAACAGGTGAAACATGATCAGCAAGTTGCTCGGATGGAAAAACTAGCTGGTTTGGTAGAAGAGCTGGAGGCTGACGAGTGGCGGTTTAAGCCCATCGAGCAGCTGCTGGGATTCACCCCCTCTTCAGGTTGATACTGCCTGGATGGTCACCTCTGGTGCGCAGCAAGTGCAAAGCCAGTGGGGGACTTTCTCACAGCTTACATAGCCATCCAGAGATCCACAGCTACGTCACTGAATTGTTAATGCACATTTGTACTTGGTTTCTCTGTATCTATTCACAGGCAACAAATACTTATATGTGTGATCTTTCAGGGAATGTTTTGTTTATTTGTTTTTAAAAGTATTGGGAATCAGATTAAGACAATCAGTTTCAGAGAACCAGGAGGTTTGGGGTTAAGAGATACTCAAAAATTTTCACAAGCCAAGTAGGGC |
| ENSG00000163516, NM_018089, NM_001042410 | ANKZF1 | GCTACAGCGTGTGCTCCATAAGCTGACCACTTTGCATGTCTATGAAGAAGACCCTCGGGAAGCAGTCAGACTGCACTCACCTCAGACACACTGGAAAACAGTAAGAGAGGAGAGAAAGAAGCCTACTGAGGAAGAAATAAGAAAGATCTGCAGGGATGAAAAGGAAGCGCTGGGGCAGAATGAGGAATCTCCCAAACAGGGTTCAGGGTCGGAGGGAGAAGATGGCTTTCAGGTAGAGTTGGAGCTAGTGGAGTTGACTGTGGGGACTCTGGATCTTTGTGAGTCTGAAGTATTGCCCAAGCGGAGGAGGAGAAAAAGGAATAAGAAGGAGAAAAGCCGAGACCAGGAGGCTGGGGCACATCGGACTCTTCTCCAGCAAACTCAAGAAGAGGAGCCTTCCA |
| ENSG00000275832 | ARHGAP23 | GGAGCTACAGCCCATCATTCCAGCGCCGGACCGGCCTCCTCCATGCGCTCTCCTTCCGGGACTCACCCTTTGGGGGGCTGCCTACCTTCAACCTGGCCCAGTCCCCTGCGTCATTCCCACCAGAGGCCTCCGAGCCACCCAGGGTTGTACGGCCGGAACCCAGCACCCGGGCCCTGGAGCCTCCTGCGGAGGATCGCGGCGATGAGGTGGTCCTGAGGCAGAAGCCCCCGACGGGCCGCAAGGTTCAGCTGACCCCCGCAAGACAGATGAACCTTGGATTTGGTGACGAGTCCCCAGAGCCAGAGGCCAGTGGGCGAGGGGAACGCCTGGGCAGGAAGGTGGCCCCTTTGGCCACCACCGAAGACTCTCTGGCTTCCA |
| ENSG00000214694, NM_001145451 | ARHGEF33 | CCTAGCCTCCGAACTCAAAACTGGTTTCACAGAAGCAATGCAAGAACTGTCAAGAATTCAACATGGAGAATATGCTTTGGAAGAAAAGGTTAAGAGCTGCAGATGTTCCATGGAAGAAAAAGTTACTGAGATGAAGAATTCATTAAACTATTTCAAGGAAGAGCTGAGCAATGCCATGTCGATGATCCAAGCCATCACTTCCAAACAAGAAGAAATGCAACAGAAAATCGAGCAGCTTCAACAGGAGAAGCGAAGAGAATCTCGAAAAGTTAAAGCCAAGAAAACTCAAAAAGAAGAGCACAGCTCACAGGCCGGGCCTGCCCAAGCACAAGGAAGTCCTTTTCGTTCTATCAATATCCCTGAGCCTGTTCTTCCAAGCGAAGACTTTACCAACCTTTTGCCTTCTCAGG |
| ENSG00000116017, NM_005224 | ARID3A | TCCTTGACCTGTTCATGCTGTACGTGCTGGTGACGGAGAAGGGCGGCCTCGTGGAGGTCATCAACAAGAAGCTGTGGCGTGAGATCACCAAGGGCCTCAACCTGCCCACGTCCATCACCAGTGCAGCCTTCACCCTGCGGACCCAATACATGAAGTACCTGTACCCCTACGAGTGTGAGAAGCGGGGCCTCAGTAACCCCAATGAGCTCCAGGCAGCCATAGACAGCAACCGACGGGAGGGCCGGCGCCAGAGCTTTGGTGGCTCCCTCTTTGCCTACTCGCCAGGCGGGGCACACGGCATGCTCTCCTCACCCAAGCTACCCGTGTCCTCCCTGGGCCTGGCCGCAAGCACCAATGGCAGCTCCATCACCCCCGCCCCTAAGATCAAGAAAGAGGAGGACTCAGCCA |
| ENSG00000047249, NM_213619, NM_213620, NM_015941 | ATP6V1H | GCAGATGGGGTAAATTGCATAATGGGAGTGTTGAGTAACAAGTGTGGCTTTCAGCTCCAGTATCAAATGATTTTTTCAATATGGCTCCTGGCATTCAGTCCTCAAATGTGTGAACACCTGCGGCGCTATAATATCATTCCAGTTCTGTCTGATATCCTTCAGGAGTCTGTCAAAGAGAAAGTAACAAGAATCATTCTTGCAGCATTTCGTAACTTTTTAGAAAAATCAACTGAAAGAGAAACTCGCCAAGAATATGCCCTGGCTATGATTCAGTGCAAAGTTCTGAAACAGTTGGAGAACTTGGAACAGCAGAAGTACGATGATGAAGATATCAGCGAAGATATCAAATTTCTTTTGGAAAAACTTGGAGAGAGTGTCCAGGACCTTAGTTCATTTGATGAATACAGTTCAGAACTTAAATCTGGAAGGTTGGAATGGAG |
| ENSG00000085224, NM_000489, NM_138270 | ATRX | CAAGGAAGAAGTGGGCTGAAGAATTTAATGATGAAACTAATGTGAGAGGACGATTATTTATCATTTCTACTAAAGCAGGATCTCTAGGAATTAATCTGGTAGCTGCTAATCGAGTAATTATATTCGACGCTTCTTGGAATCCATCTTATGACATCCAGAGTATATTCAGAGTTTATCGCTTTGGACAAACTAAGCCTGTTTATGTATATAGGTTCTTAGCTCAGGGAACCATGGAAGATAAGATTTATGATCGGCAAGTAACTAAGCAGTCACTGTCTTTTCGAGTTGTTGATCAGCAGCAGGTGGAGCGTCATTTTACTATGAATGAGCTTACTGAACTTTATACTTTTGAGCCAGACTTATTAGATGACCCTAATTCAGAAAAGAAGAAGAAGAGGGATACTCCCATGCTGCCAAAG |
| ENSG00000196366 | C9orf163 | TTTTCAACTTCCTTAGTCTCTGAGCATCAGGTCTTCATCTCTAAGATAGAGCAAGATGAGCACCTAAACGTTTCAGGGCTGAAATGGGGTTGCCAGGACAACAAGCATGGCAGGGGCGAATGGGGACATCAGGTGACTGACTGCGAGCCAGCAGAGGCCTCCACCCAGTGCTGGGAGGTGTCCCGCCGGTGGTGGCCATCACTATTCCTGGTGGGGCTTTGCTGCTTTTGCCTGCAGCATCCGCTGGTTTCTCCATTCTCCAAGTCTCTGCCCCTGCTCACACCTGGAGGCTGGACACAAGTGTCACCACTTCCCTG |
| ENSG00000135932, NM_016289 | CAB39 | TCAGATGCATTTGCCACATTCAAGGATTTACTTACAAGACATAAATTGCTCAGTGCAGAATTTTTGGAACAGCATTATGATAGATTTTTCAGTGAATATGAGAAGTTACTTCATTCAGAAAATTATGTGACAAAAAGACAGTCACTGAAGCTTCTCGGTGAACTACTACTAGATAGACACAACTTCACAATTATGACAAAATACATCAGTAAACCTGAGAACCTCAAATTAATGATGAACCTGCTGCGAGACAAAAGTCGCAACATCCAGTTTGAGGCCTTTCACGTTTTTAAGGTGTTTGTAGCCAATCCTAACAAGACGCAGCCCATCCTAGACATCCTCCTCAAGAACCAGGCCAAACTCATAGAGTTCCTCAGCAAGTTTCAGAACGACAGGACGGAGGATGAGCAGTTTAACGACGAGAAGACCTATTTAGTTAAACAGATCAGGGATTTGAAGAGACCAGCTCAGCAAGAAGC |
| ENSG00000105298, NM_001080543, NM_021231 | CACTIN | GAGGACCTGATCCAGCAGAGCCTGGACGACTACGACGCCGGCAGGTACAGCCCGCGGCTGCTCACGGCGCACGAGCTGCCACTGGACGCGCACGTGCTGGAACCGGATGAGGACCTGCAGCGCCTGCAGCTCTCGCGCCAGCAGCTCCAGGTCACGGGAGACGCCAGCGAGAGCGCCGAGGACATCTTCTTCCGGCGGGCCAAGGAGGGCATGGGCCAGGACGAGGCGCAGTTCAGCGTGGAGATGCCACTCACCGGCAAGGCCTACCTGTGGGCCGACAAGTACCGGCCACGCAAGCCGCGCTTCTTCAACCGCGTGCACACGGGCTTCGAGTGGAACAAGTACAACCAGACGCACTACGACTTTGACAACCCACCGCCCAAGATCGTGCAGGGATACAAGTTCAACATCTTCTACCCCGACCTCA |
| ENSG00000136682, ENSG00000172785, ENSG00000147996, ENSG00000204778, NM_001024916 | CBWD2 CBWD1 CBWD5 CBWD4P | CCAGGAACACAACCTCACCTTGATCAGAGTATTGTTACAATCACATTTGAAGTACCAGGAAATGCAAAGGAAGAACATCTTAATATGTTTATTCAGAATCTCCTGTGGGAAAAGAATGTGAGAAACAAGGACAATCACTGCATGGAGGTCATAAGGCTGAAGGGATTGGTGTCAATCAAAGACAAATCACAACAAGTGATTGTCCAGGGTGTCCATGAGCTCTATGATCTGGAGGAGACTCCAGTGAGCTGGAAGG |
| ENSG00000134057, NM_031966 | CCNB1 | TTGGTGTCACTGCCATGTTTATTGCAAGCAAATATGAAGAAATGTACCCTCCAGAAATTGGTGACTTTGCTTTTGTGACTGACAACACTTATACTAAGCACCAAATCAGACAGATGGAAATGAAGATTCTAAGAGCTTTAAACTTTGGTCTGGGTCGGCCTCTACCTTTGCACTTCCTTCGGAGAGCATCTAAGATTGGAGAGGTTGATGTCGAGCAACATACTTTGGCCAAATACCTGATGGAACTAACTATGTTGGACTATGACATGGTGCACTTTCCTCCTTCTCAAATTGCAGCAGGAGCTTTTTGCTTAGCACTGAAAATTCTGGATAATGGTGAATGGACACCAACTCTACAACATTACCTGTCATATACTGAAGAATCTCTTCTTCCAGTTATGCAGCACCTGGCTAAGAATGTAGTCATGGTAAATCAAGGACTTACAAAGCACATGACTGTCAAGAACAAGTATGCCACATCGAAGCATG |
| ENSG00000204345 | CD300LD | ACCTACTTGAAGTGGCGGTGTCAAGGAGCTGATTGGAATTACTGTAACATCCTTGTTAAAACAAATGGATCAGAGCAGGAGGTAAAGAAGAATCGAGTTTCCATCAGGGACAATCAGAAAAACCACGTGTTCACCGTGACCATGGAGAATCTCAAAAGAGATGATGCTGACAGTTATTGGTGTGGGACTGAGAGACCTGGAATTGATCTTGGGGTCAA |
| ENSG00000161649, NM_145273 | CD300LG | TGCAAAACTTGGAAAGATGGAGGAGAAAAAGAAAAGGAAGAAAAAAATGTCACCCATAGTCTCACCAGAGACTATCATTATTTCGTTTTGTTGTACTTCCTTCCACTCTTTTCTTCTTCACATAATTTGCCGGTGTTCTTTTTACAGAGCAATTATCTTGTATATACAACTTTGTATCCTGCCTTTTCCACCTTATCGTTCCATCA |
| ENSG00000149654 | CDH22 | GCGGGATTCTGACCAACGGCATTAAAACTGAGGCGAGACCGGGCACGGTGTGGCTCTGGGGTTAGAATGGGAGATGGGGGTGGCGTTGCAGAGTCGGGAAGGGGCGGGTCACTCAATCCTGGCCTGGGGGAGAATGCTGGAGGGAGCACGCCGCTGAGATGCCCCCACCCCAGGTTTCCCCCATCAGAGTTAAGAGGAAAGAAGGCTGTTCACTTACTAAGCACCTACTGTGTGCTGGGACGCTGTACAGAGACCAGCTCAGTCGTCAGAGAAACCATGAGGTGGTGTCCTGCACGGAATAGAAGGGGAAAGGACCC |
| ENSG00000189143 | CLDN4 | CCTGCCCTGTGCTTCATTAGCCGGTCAACAGATCCATCTCAAATACCTCCCATGGGTACTCACTGATTGCTTTAACCCAAACCATGGCACTCTTGAAGACTTTCCCTCAGGAAGCTCAAGGACTATGCATCCTTCTGGGTCAGAACTGGACACACAGCCACCAGTGCTGGACAATGGCGGCGGCTCAGGGACACACTGGAGCCCTGGCCCCTGCAGAGCTCCCAGCATGGTTGGGAAGAGAGATGCAAAATGACCACACGGCGGGTGAGGAGGAGCTCCCTCGGTGCGGCTGGGATGAGCCCTAGACACTCTCAATCACCCCCACGATGACCCCTTCCCAGAGGTCCCCTCAGTCATCTGCCCTGAACCAAGCTCTTCCTGATCCTAGACCCTCCACCCTCCCTCTATCTTCCAGGGCTTGGTGACATTCCAGGCAGAAATTTCTGACCCTTTTACTTTGGTCCCTCCCTCCCCAGCCCAGTCTCTGGTCAAACTGGATTCCTGGCTGTTC |
| ENSG00000118432, NM_016083, NM_033181 | CNR1 | CGTCTGAGGATGGGAAGGTACAGGTGACCCGGCCAGACCAAGCCCGCATGGACATTAGGTTAGCCAAGACCCTGGTCCTGATCCTGGTGGTGTTGATCATCTGCTGGGGCCCTCTGCTTGCAATCATGGTGTATGATGTCTTTGGGAAGATGAACAAGCTCATTAAGACGGTGTTTGCATTCTGCAGTATGCTCTGCCTGCTGAACTCCACCGTGAACCCCATCATCTATGCTCTGAGGAGTAAGGACCTGCGACACGCTTTCCGGAGCATGTTTCCCTCTTGTGAAGGCACTGCGCAGCCTCTGGATAACAGCATGGGGGACTCGGACTGCCTGCACAAACACGCAAACAATGCAGCCAGTGTTCACAGGGCCGCAGAAAGCTGCATCAAGAGCACGGTCAAGA |
| ENSG00000121022, NM_006837 | COPS5 | GGCACTGAAACCCGAGTAAATGCTCAGGCTGCTGCATATGAATACATGGCTGCATACATAGAAAATGCAAAACAGGTTGGCCGCCTTGAAAATGCAATCGGGTGGTATCATAGCCACCCTGGCTATGGCTGCTGGCTTTCTGGGATTGATGTTAGTACTCAGATGCTCAATCAGCAGTTCCAGGAACCATTTGTAGCAGTGGTGATTGATCCAACAAGAACAATATCCGCAGGGAAAGTGAATCTTGGCGCCTTTAGGACATACCCAAAGGGCTACAAACCTCCTGATGAAGGACCTTCTGAGTACCAGACTATTCCACTTAATAAAATAGAAGATTTTGGTGTACACTGCAAACAATATTATGCCTTAGAAGTCTCATATTTCAAATCCTCTTTGGATCGC |
| ENSG00000100056, NM_022719 | DGCR14 | ATGGAGACTTGGAACGGATGCGCCAGATTGCCATCAAGTTTGGCTCTGCCTTGGGCAAGATGTCCCGGGAGCCCCCGCCACCCTATGTGACTCCAGCCACATTTGAAACCCCTGAGGTGCATGCAGGCACTGGAGTGGTGGGCAACAAGCCCAGGCCCCGCGGCCGAGGCCTGGAGGATGGAGAGGCTGGAGAGGAGGAGGAGAAGGAGCCGCTGCCCAGCCTAGATGTCTTCCTGAGCCGCTACACGAGTGAGGACAATGCCTCCTTCCAGGAGATCATGGAGGTGGCCAAGGAGAGAAGCCGGGCACGCCACGCTTGGCTCTACCAGGCTGAGGAAGAGTTTGAGAAGAGGCAGAAAGATAATCTCGAACTCCCGTCAGCAGAGCACCAGGCCATCGAGAGCAGCCAGGCCAGTGTGGAGACCTGGAAGTACAAGGCCAA |
| ENSG00000203734, NM_001077706 | ECT2L | TTCTGCGAATCGAAGCTACAGACGTGGTCCAGCTTCACAGACTTCCTAGAAGAAACCTTGAAAACAGTAAGGAAGCAGCTGTATCCTTTCTTCAAGGAACTGCAGAAGAGCATCAGTGGCAGGATGATAGGGCAGTTTATGTTTGACACCATGGGTATGACCAACATTCTAAACAACCAAGATACTGCGCAAGCTCTGGCAGATGGATTGATGGAGTTGTCAAAAGAAGATTCTGAAAGAAATGTTGTAGAAGACAATTCTTGGGACACAAAGTCCAGGCTCAGCAAAAATGATTTAAATTTTGAAGCACTGATTAATCTGGAGAGAATACTCCAGAAGGACTCAGCAGAAAAGCGAGCTAGAGTTGTCAGAGAACTCTTACAGAGTGAGAGAAAATACGTGCAGATACTGGAAATTGTGAGAGATGTTTATGTCGCACCACTGAAAGC |
| ENSG00000165837 | ERICH6B | AAAACAATAATCAAAGAAATGGCTGCTCACAATGAACTGGAAGAGGATTTTGACATTCCCCTAACTAAGCTACTGGAAAGTGAAAACAGATGGAAACTGGTAATTATGCTGAAGAAAAATTATGAAAAGTTCAAGGAAACAATCTTACGGATTAAGAGGAGACGTGAAGCTCAAAAGTTAACAGAGATGACCAGTTTCACATTTCATTTAATGAGCAAACCAACACCTGAGAAGCCTGAGACAGAAGAAATCCAAAAGCCTCAACGTGTTGTTCATCATAGGAAGAAATTAGAACGAGATAAGGAATGGATACAGAA |
| ENSG00000070367 | EXOC5 | GTTGCACCTAATTGCCCAAGAGTTACCTTTTGATTTTTCAGAAGTTAAATCCAAAATTGCAAGTAAATACCATGATTTAGAATGCCAGCTGATTCAGGAGTTTACCAGTGCTCAAAGAAGAGGTGAAATCTCCAGAATGAGAGAAGTAGCAGCAGTTTTACTTCATTTTAAGGGTTATTCCCATTGTGTTGATGTTTATATAAAGCAGTGCCAGGAGGGTGCTTATTTGAGAAATGATATATTTGAAGACGCTGGAATACTCTGTCAAAGAGTGAACAAACAAGTTGGAGATATCTTCAGTAATCCAGAAACAGTCCTGGCTAAACTTATTCAAAATGTATTTGAAATCAAACTACAGAGTTTTGTGAAAGAGCAGTTAGAAGAATGTAGGAAGTCCGATGCAGAGCAAT |
| ENSG00000169018, NM_015322 | FEM1B | AGCTGATAAAATGGCGTGCTGCTATAGTAGTGAATGGCCATGGGATGACGCCATTGAAAGTAGCTGCCGAAAGCTGTAAAGCTGATGTCGTAGAACTGTTACTCTCTCATGCTGATTGCGACCGAAGAAGTCGGATTGAAGCTTTGGAACTCTTGGGTGCCTCCTTTGCAAATGACCGTGAGAACTATGACATCATAAAGACATACCACTATCTATATTTAGCCATGTTAGAGAGGTTCCAAGATGGTGATAACATTCTCGAAAAAGAGGTTCTTCCACCAATCCATGCTTATGGGAATAGAACTGAATGTAGAAATCCTCAGGAACTGGAGTCCATTCGGCAAGACAGAGATGCTCTTCATATGGAAGGCCTTATAGTTCGGGAACGGATTTTAGGTGCTGACAATATTGATGTTTCTCATCCCATCATTTACAGAGGAGCTGTTTATGCGGA |
| ENSG00000156466 | GDF6 | TTTAAAAAGTTTTAGTTTTTCCTAAGTGATTTTGCTCTCTTCCAATCTAAACCTGTTGCTTGTTTGGTTCAGAGAACTACAAACTGTCAAAGAAAGGGTGGGGATGATAAGAAATGCTAATATAAAAATGCTAAGTGAAAAAAAGACTTGGCCAGGAGAAATAATTTAAAATGCACATTTGCTTTGGATGCACTGTTGTTCTGTTAAGGCTGTATATATTTGTTTATTTAAGGTGACTGAAAGTGCAAAGAGGAAATGGACAGCATGCAATTCATCCTAATGTACAAAACGTTATATGCACTCAAATGTTATAATTT |
| ENSG00000049239, NM_004285 | H6PD | TAATCCTGCTGGGAGCAACTGGGGACCTGGCTAAGAAGTACTTATGGCAGGGACTGTTCCAGCTGTACCTGGATGAAGCGGGGAGGGGTCACAGTTTTAGCTTCCATGGAGCTGCTCTGACAGCCCCCAAGCAGGGTCAAGAGCTCATGGCCAAGGCCCTGGAATCCCTCTCCTGCCCCAAGGACATGGCACCCAGTCACTGTGCAGAGCACAAGGATCAGTTCCTGCAGCTGAGCCAGTACCGCCAACTGAAGACGGCCGAGGACTATCAGGCCCTGAACAAGGACATCGAGGCACAGCTCCAGCACGCAGGCCTCCGGGAGGCTGGCAGGATCTTCTACTTCTCAGTGCCACCCTTCGCCTATGAAGACATTGCCCGCAACATCAACAGTAGCTGCCGGCCAGGCCCGGGCGCCTGGCTGCGGGTTGTCCTTGAGAAACCCTTTGGCCATGACCACTTCT |
| ENSG00000166189, NM_024747 | HPS6 | CCCTGCTTCGAAGTGAAATCTTCAAACTGCTGCTGGCCGAGTTTGCCCAGCACCGCCGGCTTGATGCTCACCTCCCCCTCCTTTGCCGCCTGTGCCCACCAGAACTGGCTCCAGCTGAGCTCCTGCTTCTACTGAGGACATACCTCCCAGATGAGGTGGGGCCCCCAACCCCATTCCCTGAGCCTGGAGCAGAGCCCCCTCTCACTGTGGGCTTGCTCAAAGCCCTGCTGGAGCAGACTGGGGCTCAAGGATGGCTGTCGGGCCCAGTTCTAAGCCCATATGAGGACATCCTATGGGACCCCAGCACTCCACCCCCGACTCCACCTCGGGACCTATGACTACCCTTCAGGCATCAGAACACTCAGGGCCTGGAGGCTTGCTTGGGACTGGAGGCTTGCTTGGACAGTTCCTC |
| ENSG00000164270, NM_001040173, NM_001040172, NM_199453, NM_000870, NM_001040169 | HTR4 | GCTCTGTGGTGGCCTTCTACATCCCATTTCTCCTCATGGTGCTGGCCTATTACCGCATCTATGTCACAGCTAAGGAGCATGCCCATCAGATCCAGATGTTACAACGGGCAGGAGCCTCCTCCGAGAGCAGGCCTCAGTCGGCAGACCAGCATAGCACTCATCGCATGAGGACAGAGACCAAAGCAGCCAAGACCCTGTGCATCATCATGGGTTGCTTCTGCCTCTGCTGGGCACCATTCTTTGTCACCAATATTGTGGATCCTTTCATAGACTACACTGTCCCTGGGCAGGTGTGGACTGCTTTCCTCTGGCTCGGCTATATCAATTCCGGGTTGAACCCTTTTCTCTACGCCTTCTTGAATAAGTCTTTTAGACGTGCCTTCCTCATCATCCTCTGCTGTGATGATGAGC |
| ENSG00000197256, NM_015493 | KANK2 | CTGCAAGGTGGACAAACAGAACCGTGCTGGCTACAGCCCTATTATGCTCACCGCCCTGGCCACCCTGAAGACCCAGGACGACATCGAGACTGTCCTTCAGCTCTTCCGGCTTGGCAACATCAATGCCAAAGCCAGCCAGGCAGGACAGACGGCCCTGATGCTGGCCGTCAGCCACGGGCGGGTGGACGTTGTCAAAGCCCTGCTGGCCTGTGAGGCAGATGTCAACGTGCAAGATGATGACGGCTCCACGGCCCTCATGTGCGCCTGTGAGCACGGCCACAAGGAGATCGCGGGGCTGCTGCTGGCCGTGCCCAGCTGTGACATCTCACTCACAGATCGCGATGGGAGCACAGCTCTGATGGTGGCCTTGGACGCAGGGCAGAGTGAGATTGCGTCCATGCTGTA |
| ENSG00000104892, NM_177417 | KLC3 | GCTTTGGAGATCCGAGAGAAGGTCCTGGGTGCTGACCACCCAGATGTGGCCAAGCAGCTCAACAACCTGGCCCTGCTGTGCCAGAACCAGGGCAAGTTTGAGGACGTGGAGCGGCACTATGCCCGGGCCCTGAGCATCTATGAGGCACTGGGCGGGCCCCATGACCCCAACGTGGCCAAGACCAAGAACAACCTGGCCTCAGCCTACCTGAAACAGAACAAGTATCAACAAGCGGAAGAGCTGTACAAAGAAATCCTCCACAAGGAGGACCTACCCGCCCCTCTCGGTGCCCCCAACACAGGCACAGCTGGTGACGCAGAACAGGCCCTTCGCCGCAGCAGCTCACTCTCCAAGATCCGTGAGTCTATCAGGCGAGGAAGTGAGAAGCTGGTCTCCCGGCTCCGAGGCGAGGCGGCGGCAGGAGCAGCCGGAATGAAGAGAGCCATGTCA |
| ENSG00000189023, NM_001099921 | MAGEB16 | AGGCTTCCAGCAATCAAGAAGAGGAAGATAGTCCAAGCTCCTCAGAGGATACATCAGACCCCAGGAATGTGCCCGCAGATGCTCTCGACCAGAAAGTGGCTTTTTTGGTGAATTTCATGCTGCACAAGTGTCAGATGAAAAAGCCAATAACAAAGGCAGATATGTTGAAGATTATCATCAAAGATGATGAGAGCCACTTCTCTGAGATCCTCCTGAGAGCTTCTGAGCACCTAGAGATGATATTTGGCCTTGATGTGGTGGAGGTGGACCCCACCACCCATTGCTATGGCCTCTTCATCAAACTGGGCCTCACCTATGATGGGATGCTGAGTGGTGAAAAGGGTGTGCCCAAGACTGGCCTCCTGATAATTGTCCTGGGTGTGATCTTCATGAAGGGCAACCGTGCCACTGAAGAGGAAGTCTGGGAAGTGCTGAATTTGACGGGAGTATATTCTGGGAAGAAGCACTTCATCTTTGGAGAGCCCAGAATGCTCATC |
| ENSG00000103495, NM_001042539, NM_002383 | MAZ | AGAACCATGCCTGCGAGATGTGTGGCAAGGCCTTCCGCGACGTCTACCACCTGAACCGACACAAGCTGTCGCACTCGGACGAGAAGCCCTACCAGTGCCCGGTGTGCCAGCAGCGCTTCAAGCGCAAGGACCGCATGAGCTACCACGTGCGCTCACATGACGGCGCTGTGCACAAGCCCTACAACTGCTCCCACTGTGGCAAGAGCTTCTCCCGGCCGGATCACCTCAACAGTCACGTCAGACAAGTGCACTCAACAGAACGGCCCTTCAAATGTGAGAAATGTGAGGCAGCTTTCGCCACGAAGGATCGGCTGCGGGCGCACACAGTACGACACGAGGAGAAAGTGCCATGTCACGTGTGTGGCAAGATGCTGAGCTCGGCTTATATTTCGGACCACATGAAGGTGCACAG |
| ENSG00000151611, NM_172250 | MMAA | ATCAGGAATCCCATGTGCTCAGCCGTTTAATTCTCTTGGACTCCATTGTACAAAGTGGATGCTGCTGTCAGATGGCTTAAAGAGAAAATTATGTGTACAAACAACCTTAAAGGACCACACAGAAGGACTTTCTGATAAAGAGCAAAGATTTGTGGATAAACTTTATACTGGTTTAATCCAAGGGCAAAGGGCCTGTTTAGCAGAGGCCATAACTCTTGTAGAATCAACTCACAGCAGGAAAAAGGAGTTAGCCCAGGTGCTTCTTCAGAAAGTATTACTTTACCACAGAGAACAAGAACAATCAAATAAAGGAAAACCACTAGCATTTCGAGTAGGATTGTCTGGGCCCCCTGGTGCTGGAAAATCAACATTTATAGAATATTTTGGAAAAATGCTTACTGAGAGAGGGCACAAATTATCTGTGCTAGCTGTGGACCCTTCTTCTTG |
| ENSG00000114686, NM_007208 | MRPL3 | CCTGGAAAAATGGGAAACATATACAGGACAGAATATGGACTGAAAGTGTGGAGAATAAACACAAAGCACAACATAATCTATGTAAATGGCTCTGTACCTGGACATAAAAATTGCTTAGTAAAGGTCAAAGATTCTAAACTGCCTGCATATAAGGATCTCGGTAAAAATCTACCATTCCCTACATATTTTCCTGATGGAGATGAAGAGGAACTGCCAGAAGATTTGTATGATGAAAACGTGTGTCAGCCCGGTGCGCCTTCTATTACATTTGCCTAACATCTTTGGACGTGGCAGAACCTTACATATTCTGTGAGCTTCGATGAGCCAGAGTGATATCATAACCACCAGAAATCATACTCTCCTTTCTTAGTCACAACAAAATCACACATGTCATCTTTGTCAAGGGCATAAATATATCATTCATACCCCCATTAAATTTTGTTAGAAAAATTACCACATTAAATATATGAGTTAAGTAGATTGGATTTGCTGAAATTGGTGTTGGGCATATTAGCA |
| ENSG00000105364, NM_146388, NM_146387, NM_015956 | MRPL4 | TGGCAGGACTCACGTTACAGACCCCTCTACCCCTTCAGCCTGCCCTACAGCGACTTCCCCCGACCCCTACCCCACGCTACCCAGGGCCCAGCGGCCACCCCGTACCACTGTTGATGTGAAGCACCTCTTCTGAGCCAGGCCGAGCCCCTGGCCGACTTGGGAGCCTCAGGCCCACGCCCACCCTTCGAGGAAGGTGTCACCTGGACCCCTTCATTCCACGGAGGAAGCTGAGGCCACAGGGAGCGGCCATCGCCATTGGGAAGGGGCGACTCCACGGAAAGCCCAGACGGGCTTCTGCATCCATTCCCTCTTT |
| ENSG00000198015, NM_172177, NM_014050, NM_172178 | MRPL42 | CACCCAAGAATGTGCTTTCCTGGAAATTTTTCTAGATAGAATATTATGTAATTTGTTTTGAAGGTTTTTTTTCCTGACTGTCTTAAAGATTATGCTAGCTTTAGAACACATGCAAACAGCTGCAGCTCTGTGATTAAAATGTTAAGGTCACTAGAATAGTGTTAGGAAAATGTTAATAGATAATTCTGCTGCCTCTATTAGCTTATGTATGATCCTTAATAGCATTGCTTCCCCTTCAGTTTCTATTTAGGAGGTGAAGAATCTTTTAATAAGGTAGAAAGTTGCTTTATTTAAATTTGTACTGTAGAATGTTTGTGATACAGTATTTGTAAAATTGATATGAAAAATAATTTCTCAAAGTCTATTGTTTGATCTAGTTATTTTAATAAAGGAACTCTAGAAGTTTCAAGTGGCCAGTGAAGTGTGAGGAATAGAGAGTAAAGGAAATAATTAATAATGGTGACCCTCTGTTAAGTGG |
| ENSG00000136897, NM_019051 | MRPL50 | TGTGGACAGTCTCAGGGACACCATGTAGAGAATTTTGGTCTCGATTCAGAAAAGAGAAAGAGCCAGTGGTTGTTGAGACAGTAGAAGAGAAAAAGGAACCTATCCTAGTGTGTCCACCTTTACGAAGCCGAGCATACACACCACCTGAAGATCTCCAGAGTCGTTTGGAATCTTACGTTAAAGAAGTTTTTGGTTCATCTCTTCCTAGTAATTGGCAAGACATCTCCCTGGAAGATAGTCGTCTAAAGTTCAATCTTCTGGCTCATTTAGCTGATGACTTGGGTCATGTAGTCCCTAACTCCAGACTCCACCAGATGTGCAGGGTTAGAGATGTTCTTGATTTCTATAATGTCCCTATTCAAGATAGATCTAAATTTGATGAACTCAGTGCCAGTAATCTGCCCCCCAATTTGAAAATCACTTGGAGTTACTAAGCAATTCGGAAGAGAAACACATTGAAATCACTGTCTTTCCCTGAGCAAGG |
| ENSG00000159593, NM_003905, NM_001018160, NM_001018159 | NAE1 | ATCATGGGCAAGAGGCTTTAGAATCTGCTCATGTTTGCCTAATAAATGCAACAGCCACAGGAACTGAAATTCTTAAAAACTTGGTACTACCAGGTATTGGTTCGTTTACAATTATTGATGGAAATCAGGTCAGCGGAGAAGATGCTGGAAACAATTTCTTCCTTCAAAGAAGCAGTATCGGCAAGAACCGAGCTGAAGCTGCCATGGAATTCTTACAAGAATTAAATAGCGATGTCTCTGGAAGTTTTGTGGAAGAGAGTCCAGAAAACCTTCTAGACAATGATCCCTCATTTTTCTGTAGGTTTACTGTTGTAGTTGCAACTCAGCTTCCTGAAAGCACTTCACTACGCTTAGCAGATGTCCTCTGGAATTCCCAGATTCCTCTTTTGATCTGTAGGACA |
| ENSG00000224712, ENSG00000254852 | NPIPA3 NPIPA2 NPIPA8 | GGCACTCTGCTTGGGTTATCAATACTCTGGCTGACCATCGTCATCGTGGGACTGACTTTGGTGGAAGTCCTTGGTTACTTATCATTACTGTGTTTCTGAGAAGTTATAAATTTGCCATCTCCCTCTGCACAAGTTACCTTTGTGTGTCTTTCCTGAAGACTATCTTCCCGTCTCAAAATGGACATGATGGATCCACGGATGTACAGCAGAGAGCCAGGAGGTCCAACCGCCGTAGACAGGAAGGAATTAAAATTGTCCTGGAAGACATCTTTACTTTATGGAGACAGGTGGAAACCAAAGTTCGAGCTAAAATCTGTAAGATGAAGGTGACAACAAAAGTCAACCGTCATGACAAAATCAATGGAAAGAGGAAGACCGCCAAAGAACATCTGAGGAAACTAAGCATGAAAGAACGTGAGCACGGAGAAAAGGAGAGGCAGGTGTCAGAGGCAGAGGAAAACG |
| ENSG00000198400, NM_001007792, NM_002529, NM_001012331 | NTRK1 | TGCCTGCCTCTTCCTTTCTACGCTGCTCCTTGTGCTCAACAAATGTGGACGGAGAAACAAGTTTGGGATCAACCGCCCGGCTGTGCTGGCTCCAGAGGATGGGCTGGCCATGTCCCTGCATTTCATGACATTGGGTGGCAGCTCCCTGTCCCCCACCGAGGGCAAAGGCTCTGGGCTCCAAGGCCACATCATCGAGAACCCACAATACTTCAGTGATGCCTGTGTTCACCACATCAAGCGCCGGGACATCGTGCTCAAGTGGGAGCTGGGGGAGGGCGCCTTTGGGAAGGTCTTCCTTGCTGAGTGCCACAACCTCCTGCCTGAGCAGGACAAGATGCTGGTGGCTGTCAAGGCACTGAAGGAGGCGTCCGAGAGTGCTCGGCAGGACTTCCAGCGTGAGGCTG |
| ENSG00000174516 | PELI3 | CTAGCACTTGCTGATAGCCACTATGGGCTCGACTCTGTGCTAAGTGGTATCTGGGGACACAGAGGCAATCAGACCTGGTTCCCCCCCTGGTGGAGTTCACAGTCTAGTGGAGGACAGACTGTTAACAAATGGCAACACGGCTTCATGCGTGCCAGGATCAGAGTACAAATCAGGCTGTGGCAGGCCTCACTGGTAATGGAAAAGTGAGGGAGGCTTCACGGAGGAGGTGTCATAGGAGCTCAGCCTCAACGGCTGCTCGGAACCCTCCGAGTCCACAAGATGGGAAGCAAGTCCACAAGATGGGAAGCGTGCTCTAT |
| ENSG00000187583, NM_032129 | PLEKHN1 | TGTCTTCCTCCCACCAGAAGTGCCCCCAGCTTGGAGGGCCTGAGGCCAGTGGGGGGCTTGTGCAGTGGATCTGATGGCCGCGGTGAGGTGGGTTCTCAGGACCACCCTCGCCAAGCTCCAGGGTACCTGCCCCTCTAACCCACTTCAAATTACAAGTCAGGGTCTGAACCCAGTGTGATGGGGGGAGTCTCTGGGGCCCTGAGTTCAGAGCCCGTCCCTCAGCTCCTGTTCCTTGGTGCCAGCAGCTGGGGCAGGGAAGGGTGGGAGGGGCCCCATCCAAAGGATGCCCTGGCCAGCGAGGCTGGGTCACAGGTCAGGGAGGTCCTGGCCGTCCACAGGGTCGGCCCTCAGCTCAGCCCGCCAGGAGTCAGGGAGGAGACTCGCTGGGAGTGGGAGGGCAGCACGGGCGTGAAGGTCGGAGGACAGAG |
| ENSG00000235961 | PNMA6A | TCTGCTTTCCCTGCGTAGATCTAGGCCAGGGGCTGCTTGTTTTTGTGGAGCCGTGTGTGTTCTTCTCTGAGCAGCTCCTCCCCAGAGGACCCCAGCGCAGTCCCGGGAGATGGCGGAAAGAAGGCACCAGGGCACAGTGGACACTCATCCCGTGACAGCGATGGTGACCATGACTGTGGGAGAAAGAACAGGACCCGGGATGGAGTGGGGCTGTCTGAGTTTCCCCAGTGAACTTTGTGCTTTGGCGTTCCACCCCTGTTGTTAC |
| ENSG00000165916, NM_002804 | PSMC3 | AGTGACTCGGCAGGAGAAGATGGCGACCGTGTGGGATGAGGCCGAGCAAGATGGAATTGGGGAGGAGGTGCTCAAGATGTCCACGGAGGAGATCATCCAGCGCACACGGCTGCTGGACAGTGAGATCAAGATCATGAAGAGTGAAGTGTTGAGAGTCACCCATGAGCTCCAAGCCATGAAGGACAAGATAAAAGAGAACAGTGAGAAAATCAAAGTGAACAAGACCCTGCCGTACCTTGTCTCCAACGTCATCGAGCTCCTGGATGTTGATCCTAATGACCAAGAGGAGGATGGTGCCAATATTGACCTGGACTCCCAGAGGAAGGGCAAGTGTGCTGTGATCAAAACCTCTACACGACAGACGTACTTCCTTCCTGTGATTGGGTTGGTGGATGCTGAAAAGCTAAAGCCAGGAGACCTGGTG |
| ENSG00000169398, NM_153831 | PTK2 | CAATCCCACACATCTTGCTGACTTCACTCAAGTGCAAACCATTCAGTATTCAAACAGTGAAGACAAGGACAGAAAAGGAATGCTACAACTAAAAATAGCAGGTGCACCCGAGCCTCTGACAGTGACGGCACCATCCCTAACCATTGCGGAGAATATGGCTGACCTAATAGATGGGTACTGCCGGCTGGTGAATGGAACCTCGCAGTCATTTATCATCAGACCTCAGAAAGAAGGTGAACGGGCTTTGCCATCAATACCAAAGTTGGCCAACAGCGAAAAGCAAGGCATGCGGACACACGCCGTCTCTGTGTCAGAAACAGATGATTATGCTGAGATTATAGATGAAGAAGATACTTACACCATGCCCTCAACCAGGGATTATGAGATTCAAAGAGAAAGAATAGAACTTGGACGATGTATTGGAGAAGGCCAATTTGGAGATG |
| ENSG00000119707, NM_021239 | RBM25 | GCTCTGCTCCATCTGTTTCCTCTGCCAGTGGCAATGCAACACCTAACACTCCTGGGGATGAGTCTCCCTGTGGTATTATTATTCCTCATGAAAACTCACCAGATCAACAGCAACCTGAGGAGCATAGGCCAAAAATAGGACTAAGTCTTAAACTGGGTGCTTCCAATAGTCCTGGTCAGCCTAATTCTGTGAAGAGAAAGAAACTACCTGTAGATAGTGTCTTTAACAAATTTGAGGATGAAGACAGTGATGACGTACCCCGAAAAAGGAAACTGGTTCCCTTGGATTATGGTGAAGATGATAAAAATGCAACCAAAGGCACTGTAAACACTGAAGAAAAGCGTAAACACATTAAGAGTCTCATTGAGAAAATCCCTACAGCCAAACCTGAGCTCTTCGCTT |
| ENSG00000177963, NM_021932 | RIC8A | AGCGTTTGCACAAGACACACAGGCTGAAGGAGAGTGTAGCTCCCGTGCTGAGCGTGCTGACTGAATGTGCCCGGATGCACCGCCCAGCCAGGAAGTTCCTGAAGGCCCAGGGATGGCCACCTCCCCAGGTGCTGCCCCCTCTGCGGGATGTGAGGACACGGCCTGAGGTTGGGGAGATGCTGCGGAACAAGCTTGTCCGCCTCATGACACACCTGGACACAGATGTGAAGAGGGTGGCTGCCGAGTTCTTGTTTGTCCTGTGCTCTGAGAGTGTGCCCCGATTCATCAAGTACACAGGCTATGGGAATGCTGCTGGCCTTCTGGCTGCCAGGGGCCTCATGGCAGGAGGCCGGCCCGAGGGCCAGTACTCAGAGGATGAGGACACAGACACAGATGAGTACAAGGAAGCCAAAGCCAGCATAAACCCTGTGACCGGGAGGGTGGAGGAGAAGCCGCCTAACCCTATGGAGGGCAT |
| ENSG00000080345, NM_018151 | RIF1 | GTGGCCACCATGAAGACTTTGCTTAGAACTTGGTCAGAATTATATAGAGCATTTGCTCGTTGTGCTGCTTTGGTGGCAACAGCAGAAGAGAACTTGTGCTGTGAGGAACTTTCTTCCAAGATAATGTCCAGTTTGGAAGATGAAGGCTTTTCTAATTTGTTGTTCGTGGATAGAATTATTTATATTATTACTGTAATGGTTGATTGCATTGACTTCTCACCATATAATATTAAATATCAGCCCAAAGTTAAATCACCACAGAGACCTTCAGATTGGTCCAAAAAGAAGAATGAGCCCCTAGGGAAA |
| ENSG00000189051 | RNF222 | TCTGTCTAAAAAAAAAAAAAAATTACAGAAGAGCACAGGTGCTATTGGAGGGGAGGGATACAGATGGATTCCATGGGGAAAATATTTATTATAGGAATAAAAATAATGTTAAATTTGCAAAGACTCCAATGAGTCTCTTTTAACCTCAGCCAGGCTGTTTTAAACAGTGTCATGAATTTCTGGGAACCTGGTTCTAGTCTTTTCAGATTCTGTCGTATTTGCCAAAAGTTTCTTTCAACATACTCTCAGTGTTTTTGTATGTTCAATGGTGACCTGTAGCTGCAACTCCTTTTATTGTCAGAGAATGCCAAAAGCTA |
| ENSG00000089009, NM_001024662, NM_000970 | RPL6 | GGTACCCGGGTGGTTAAACTTCGCAAAATGCCTAGATATTATCCTACTGAAGATGTGCCTCGAAAGCTGTTGAGCCACGGCAAAAAACCCTTCAGTCAGCACGTGAGAAAACTGCGAGCCAGCATTACCCCCGGGACCATTCTGATCATCCTCACTGGACGCCACAGGGGCAAGAGGGTGGTTTTCCTGAAGCAGCTGGCTAGTGGCTTATTACTTGTGACTGGACCTCTGGTCCTCAATCGAGTTCCTCTACGAAGAACACACCAGAAATTTGTCATTGCCACTTCAACCAAAATCGATATCAGCAATGTAAAAATCCCAAAACATCTTACTGATGCTTACTTCAAGAAGAAGAAGCTGCGGAAGCCCAGACACCAGGAAGGTGAGATCTTCGACACAGAAAAAGAGAAATATGAGATTACGGAGCAGCGCAAGATTGATCAGAAAGCTGTGGACTCACAAATTTTACCAAAAATCAAAGCTATTCCTCAGCTCCAGGGCTACCTGCGATCTGTGTTTGC |
| ENSG00000123453, NM_001134707, NM_007101 | SARDH | GGAGGAGGAGACGGGACTACACACGGGCTGGATCCAGAATGGGGGCCTCTTCATCGCGTCCAACCGGCAGCGCCTGGACGAGTACAAGAGGCTCATGTCGCTGGGCAAGGCGTATGGTGTGGAATCCCATGTGCTGAGCCCGGCAGAGACCAAGACTCTGTACCCGCTGATGAATGTGGACGACCTCTACGGGACCCTGTATGTGCCGCACGACGGTACCATGGACCCCGCTGGCACCTGTACCACCCTCGCCAGGGCAGCTTCTGCCCGAGGAGCACAGGTCATTGAGAACTGCCCAGTGACCGGCATTCGTGTGTGGACGGATGATTTTGGGGTGCGGCGGGTCGCGGGTGTGGAGACTCAGCATGGTTCCATCCAGACACCCTGCGTGGTCAACTGTGCAGGAGTGTGG |
| ENSG00000254415, ENSG00000268500, ENSG00000105501 | SIGLEC14 AC018755.18 SIGLEC5 | CTGCAGGAGAAGCCAGTGTACGAGCTGCAAGTGCAGAAGTCGGTGACGGTGCAGGAGGGCCTGTGCGTCCTTGTGCCCTGCTCCTTCTCTTACCCCTGGAGATCCTGGTATTCCTCTCCCCCACTCTACGTCTACTGGTTCCGGGACGGGGAGATCCCATACTACGCTGAGGTTGTGGCCACAAACAACCCAGACAGAAGAGTGAAGCCAGAGACCCAGGGCCGATTCCGCCTCCTTGGGGATGTCCAGAAGAAGAACTGCTCCCTGAGCATCGGAGATGCCAGAATGGAGGACACGGGAAGCTATTTCTTCCGCGTGGAGAGAGGAAGGGATGTAAAATATAGCTACCAACAGAATAAGCTGAACTTGGAGGTGACAGCCCTGATAGAGAAACCCGACATCCACTTTCTGGAGCCTC |
| ENSG00000137648, NM_019894 | TMPRSS4 | CTGAACAGCCTCGATGTCAAACCCCTGCGCAAACCCCGTATCCCCATGGAGACCTTCAGAAAGGTGGGGATCCCCATCATCATAGCACTACTGAGCCTGGCGAGTATCATCATTGTGGTTGTCCTCATCAAGGTGATTCTGGATAAATACTACTTCCTCTGCGGGCAGCCTCTCCACTTCATCCCGAGGAAGCAGCTGTGTGACGGAGAGCTGGACTGTCCCTTG |
| ENSG00000168477, NM_019105, NM_032470, NR_001284 | TNXB | CCTGGTGGTACAGGAACTGCCACTACGCCAACCTCAACGGGCTCTACGGGAGCACAGTGGACCATCAGGGAGTGAGCTGGTACCACTGGAAGGGCTTCGAGTTCTCGGTGCCCTTCACGGAAATGAAGCTGAGACCAAGAAACTTTCGCTCCCCAGCGGGGGGAGGCTGAGCTGCTGCCCACCTCTCTCGCACCCCAGTATGACTGCCGAGCACTGAGGGGTCGCCCCGAGAGAAGAGCCAGGGTCCTTCACCACCCAGCCGCTGGAGGAAGCCTTCTCTGCCAGCGATCTCGCAGCACTGTGTTTACA |
| ENSG00000173769 | TOPAZ1 | ACCATTGGAAGGAAATTTATACCGAAAACTTCTTCTAATTCCATCTTATTTATCTGAGATTGAAATGCTCTTAGCTATTGAAATCTTCATGGTATCTAATGCTAGTAGTATTCAGAGTCCTGGAACTTCTACACAGATACTGCAGATAGTTTTGAAAAGGTGTGAAGACAACCAGTCTCGGAGCAATGATGATTATCAAGCTGCAGTAGAAAGGTTAATTATGGCTGCTCGTATATCAGATCCAAAGCTTTTCGTTAAACACATGACTGTCAATGTTAATAAGGAACAGGTTTATAGTTTGGAACATTGTTCTGCCC |
| ENSG00000186591 | UBE2H | AGCTTTTTTCAGAGAGTGACCGAATAGGAAGAAGGGATTCAGAAATATGATGTAGTAGAACATGCCCTGCAGGAAGAAAATGTTTTCTCTCCTGCGTATGTTAAGAGGAAATCAGTTTAAAATGCCTGCTAATCAAATGTCGACTCAGCTAACCATATGTCCAGGTTGCTGCAGAGTTTCTTTCAAATCTGAGTGTTGCCTGCTGAACTCGGAGGGGAGTTTGGAGAAATTTTAGACAGCAGCTCTGTGTTCTTTTTTTGCCGCAGCCAATAAGATAATAGCAGAATTAGCCAATCCTAAAACCTTCTAAAGACGAT |
| ENSG00000165280, NM_007126 | VCP | TGAAGCCATCAATGAGGACAACAGTGTGGTGTCCTTGTCCCAGCCCAAGATGGATGAATTGCAGTTGTTCCGAGGTGACACAGTGTTGCTGAAAGGAAAGAAGAGACGAGAAGCTGTTTGCATCGTCCTTTCTGATGATACTTGTTCTGATGAGAAGATTCGGATGAATAGAGTTGTTCGGAATAACCTTCGTGTACGCCTAGGGGATGTCATCAGCATCCAGCCATGCCCTGATGTGAAGTACGGCAAACGTATCCATGTGCTGCCCATTGATGACACAGTGGAAGGCATTACTGGTAATCTCTTCGAGGTATACCTTAAGCCGTACTTCCTGGAAGCGTATCGACCCATCCGGAAAGGAGACATTTTTCTTGTCCGTGGTGGGATGCGTGCTGTGGAGTTCAAAGTGG |
| ENSG00000172967, NM_175878 | XKR3 | CTTGGCCAGAGACTCCATCTAAGCTTTCCTTTTAGCATTATCTTCTCAACTGTTCTCTACTGTGGTGAGGTTGCCTTTGGTTTATACATGTTTGAAATTTATCGAAAAGCTAATGACACATTCTGGATGTCATTTACCATCAGCTTTATTATTGTGGGGGCAATTTTGGATCAAATTATCCTGATGTTTTTCAACAAAGACTTGAGGAGAAATAAGGCTGCATTACTTTTTTGGCACATTCTTCTTTTAGGACCTATTGTGAGGTGTTTGCACACCATTAGAAATTACCACAAATGGTTGAAAAATCTTAAACAGGAGAAGGAAGAGACTCAAGTTAGCATCACAAAGAGAAACACGATGCTGGAAAGGGAGATTGCATTCTCAATCCGGGATAATTTCATGCAGCAGAAGGCT |
| ENSG00000196453, NM_015694 | ZNF777 | ATGTTGCTGTGCACTTCTCGGAGCAGGAGTGGGGAAACCTGTCTGAGTGGCAGAAGGAGCTCTACAAGAACGTGATGAGGGGCAACTACGAGTCCCTGGTTTCCATGGACTATGCAATTTCCAAACCAGACCTCATGTCACAGATGGAGCGCGGGGAGCGGCCCACCATGCAGGAGCAGGAAGACTCTGAGGAGGGCGAAACGCCGACAGATCCCAGTGCTGCGCACGATGGGATCGTGATTAAGATCGAGGTACAGACCAACGACGAGGGCTCAGAAAGTTTGGAGACACCTGAGCCCCTGATGGGACAGGTGGAAGAGCACGGCTTCCAGGACTCAGAGCTGGGTGACCCCTGTGGGGAACAGCCAGACCTGGACATGCAGGAGCCAGAGAACACGCTGGAGGAGTC |

**Supplementary Table 6. Our custom-made esiRNA library.**
