# Supplementary material for: Towards a data-integrated cell
Source: Nat Commun. 2019 Feb 18;10:805. doi: 10.1038/s41467-019-08797-8 (PMC6379402; doi:10.1038/s41467-019-08797-8)
Supplement: Supplementary file 3 — Reporting Summary [file 41467_2019_8797_MOESM3_ESM.pdf]

nature research

Corresponding author(s): N. Malod-Dognin and N. Przulj

Last updated by author(s): Jan 17, 2019

Reporting Summary

Nature Research wishes to improve the reproducibility of the work that we publish. This form provides structure for consistency and transparency in reporting. For further information on Nature Research policies, see Authors & Referees and the Editorial Policy Checklist.

Statistics

For all statistical analyses, confirm that the following items are present in the figure legend, table legend, main text, or Methods section.

n/a	Confirmed	
☐	☒	The exact sample size (*n*) for each experimental group/condition, given as a discrete number and unit of measurement
☐	☒	A statement on whether measurements were taken from distinct samples or whether the same sample was measured repeatedly
☐	☒	The statistical test(s) used AND whether they are one- or two-sided<br>*Only common tests should be described solely by name; describe more complex techniques in the Methods section.*
☐	☒	A description of all covariates tested
☐	☒	A description of any assumptions or corrections, such as tests of normality and adjustment for multiple comparisons
☐	☒	A full description of the statistical parameters including central tendency (e.g. means) or other basic estimates (e.g. regression coefficient) AND variation (e.g. standard deviation) or associated estimates of uncertainty (e.g. confidence intervals)
☐	☒	For null hypothesis testing, the test statistic (e.g. *F*, *t*, *r*) with confidence intervals, effect sizes, degrees of freedom and *P* value noted<br>*Give P values as exact values whenever suitable.*
☒	☐	For Bayesian analysis, information on the choice of priors and Markov chain Monte Carlo settings
☒	☐	For hierarchical and complex designs, identification of the appropriate level for tests and full reporting of outcomes
☐	☒	Estimates of effect sizes (e.g. Cohen's *d*, Pearson's *r*), indicating how they were calculated

Our web collection on statistics for biologists contains articles on many of the points above.

Software and code

Policy information about availability of computer code

Data collection	No software was used. Data was collected manually from public databases
Data analysis	Tissue-specific networks are created using a custom Python (2.7) script using NetworkX (1.1) library<br>- iCell data integration is performed using a custom Matlab (2014a) script<br>- Topological signatures of nodes in iCell and in the other networks are computed using the open source sorftware ORCA (http://www.biolab.si/supp/orca).<br>- Clustering and enrichment analyses are performed using dedicated Python (2.7) script using Scipy (0.18.1) and sklearn libraries<br>- Identification of rewired genes in cancer is done using a custom Python (2.7) script.<br>- All plots are generated using python (2.7) and matplotlib (2.0.0) libraries.<br>- Differential expression of genes are computed using GEPIA web-server (http://gepia.cancer-pku.cn/)

For manuscripts utilizing custom algorithms or software that are central to the research but not yet described in published literature, software must be made available to editors/reviewers. We strongly encourage code deposition in a community repository (e.g. GitHub). See the Nature Research guidelines for submitting code & software for further information.

Data

Policy information about availability of data

All manuscripts must include a data availability statement. This statement should provide the following information, where applicable:

- Accession codes, unique identifiers, or web links for publicly available datasets
- A list of figures that have associated raw data
- A description of any restrictions on data availability

Data and software generated during the study are publicly available at http://www0.cs.ucl.ac.uk/staff/natasa/iCell

Field-specific reporting

Please select the one below that is the best fit for your research. If you are not sure, read the appropriate sections before making your selection.

☒ Life sciences ☐ Behavioural & social sciences ☐ Ecological, evolutionary & environmental sciences

For a reference copy of the document with all sections, see nature.com/documents/nr-reporting-summary-flat.pdf

Life sciences study design

All studies must disclose on these points even when the disclosure is negative.

Sample size	For the two types of experiments that are subject to sample sizes (the survival curve analyses and the differential gene expression analyses), we used all the samples that were availlable from the corresponding TCGA projects, which makes our study as comprehensive as possible.
Data exclusions	For the two above-mentionned experiments, no data was excluded.
Replication	The only experiment that can be subject to experimental noise is our knock-down & cell growth experiment (in which we tested if our newly uncovered genes can influence the growth of cancer cell lines). This experiment was standardly performed in triplicate (means and variances over the triplicate are reported in the paper and the supplement).
Randomization	N/A
Blinding	N/A

Reporting for specific materials, systems and methods

We require information from authors about some types of materials, experimental systems and methods used in many studies. Here, indicate whether each material, system or method listed is relevant to your study. If you are not sure if a list item applies to your research, read the appropriate section before selecting a response.

Materials & experimental systems

n/a	Involved in the study	
☒	☐	Antibodies
☐	☒	Eukaryotic cell lines
☒	☐	Palaeontology
☒	☐	Animals and other organisms
☒	☐	Human research participants
☒	☐	Clinical data

Methods

n/a	Involved in the study	
☒	☐	ChIP-seq
☒	☐	Flow cytometry
☒	☐	MRI-based neuroimaging

Eukaryotic cell lines

Policy information about cell lines

Cell line source(s)	Cell lines used in our study were obtained from ATCC (CRL-1831) or from the existing lab stocks, which originally were purchased from ATCC and further cultivated (not more than 20 passages).<br>The following cell lines were used in this study:<br>• MCF7 (ATCC® HTB-22) breast cancer cell line (tissue: breast mammary gland, cell type: epithelial, disease: adenocarcinoma)<br>• HCT116 (ATCC® CCL-247) colon cancer line (tissue: colon, cell type: epithelial, disease: colorectal carcinoma)<br>• A549 (ATCC® CRM-CCL-185) lung cancer line (tissue: lung, cell type: epithelial, disease: carcinoma)<br>• PC-3 (PC-3 ATCC ® CRL-1435™) prostate cancer line (tissue: prostate, cell type: epithelial, disease type: grade IV, adenocarcinoma)
Authentication	Cell lines were purchased directly from ATCC and don't need to be authenticated, thus we have not done it in this case.
Mycoplasma contamination	ATCC cell lines are provided with the assurance that they are negative for mycoplasma. Furthermore, we regularly check for mycoplasma all cell lines that are cultured for a long-term period in the lab.
Commonly misidentified lines<br>(See ICLAC register)	N/A
